# Development of a 3D Human Colon Model Along with Bioelectronics for the Induction and Monitoring of Diseases

**DOI:** 10.1002/advs.202506377

**Published:** 2025-09-27

**Authors:** Jorge Alfonso Tavares‐Negrete, Sahar Najafikoshnoo, Anita Ghandehari, Mozhgan Keshavarz, Quinton Smith, Armand Ahmetaj, Steven Zanganeh, Rahim Esfandyarpour

**Affiliations:** ^1^ Department of Biomedical Engineering University of California Irvine CA 92697 USA; ^2^ Henry Samueli School of Engineering University of California Irvine CA 92697 USA; ^3^ Laboratory for Integrated Nano Bio Electronics Innovation The Henry Samueli School of Engineering University of California Irvine CA 92697 USA; ^4^ Department of Electrical Engineering and Computer Science University of California Irvine CA 92697 USA; ^5^ Sue and Bill Gross Stem Cell Research Center University of California Irvine CA 92697 USA; ^6^ Department of Chemical and Biomolecular Engineering University of California Irvine CA 92697 USA; ^7^ Bioengineering Program New York Institute of Technology New York NY 11568 USA; ^8^ Biomedical Research, Innovation, and Imaging Center (BRIIC) New York Institute of Technology New York NY 11568 USA; ^9^ Department of Mechanical and Aerospace Engineering University of California Irvine CA 92697 USA

**Keywords:** 3D colon human model, colorectal cancer modeling, in vivo mimicking, non‐animal model

## Abstract

Conventional in vitro and animal models do not reproduce the geometry, mechanics, or transport physics of the human colon, limiting their fidelity for disease studies and drug screening. A patient‐derived, freeform reversible embedding of suspended hydrogels bioprinted three‐dimensional (3D) in vivo mimicking human‐colon model (3D‐IVM‐HC) is reported whose micro‐computed tomography (CT) profile deviates by less than 4% from the original computed tomography template and spontaneously forms crypt‐like invaginations with a median depth of 65 µm. The dual‐layer gelatin methacrylate (GelMA)/alginate matrix matches native colonic stiffness (9–65 kPa) and sustains >95% cell viability with a 14‐fold metabolic increase over 14 days. Caco‐2 epithelia polarize within the lumen, form continuous Zonula occludens‐1 (ZO‐1) belts, and reach a transepithelial electrical resistance (TEER) of 68 ± 4 Ω cm^2^, values within the human ex vivo range. Finite‐element simulations (FEM) parameterized with measured geometry and resistance predict water and nutrient fluxes within 80–99% of human explants. When HCT116 tumor spheroids are introduced, the construct yields a 5‐fluorouracil (5‐FU) half‐maximal inhibitory concentration (IC_5_₀) of 540 ± 30 µm, an order of magnitude higher than a matched two‐dimensional (2D) monolayer (42 ± 5 µm), mirroring clinical chemoresistance. Together, these benchmarks establish the 3D‐IVM‐HC as a physiologically faithful, non‐animal model for probing colorectal biology and quantifying drug response.

## Graphical abstract

We present a functional three‐dimensional in‐vivo‐mimicking human colon (3D‐IVM‐HC) model engineered via Freeform Reversible Embedding of Suspended Hydrogels bioprinting with gelatin methacrylate (GelMA)/alginate hydrogels. This 3D non‐animal model replicates crypt‐like architectures, epithelial polarization, and stromal–tumor interactions, showing clinically relevant chemoresistance to 5‐fluorouracil. The 3D‐IVM‐HC enables physiologically accurate, animal‐free colorectal disease modeling and precision drug discovery.

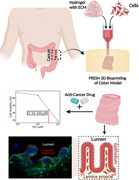

## 1 Introduction

To effectively investigate disease progression mechanisms and rigorously evaluate drug efficacy, researchers have traditionally relied upon various experimental models, predominantly animal‐based systems,^[^ ^1^, ^2^, ^3^ ^]^ due to their biological similarities with human physiology.^[^ ^4^, ^5^ ^]^ However, these conventional animal models present significant translational limitations arising from inherent species‐specific physiological differences,^[^ ^5^ ^]^ fundamentally compromising their predictive accuracy and clinical relevance.^[^ ^5^, ^6^, ^7^, ^8^ ^]^ For instance, a seminal analysis presented by Olson et al. revealed that approximately half of rodent‐derived toxicological findings fail to accurately predict human toxicities, emphasizing the profound challenges of extrapolating animal‐derived data to human clinical outcomes.^[^ ^7^ ^]^ Further complicating their utility, animal models often inadequately capture critical aspects of human tumor biology, such as the extensive tumor heterogeneity, intricate microenvironment interactions, and dynamic disease progression observed clinically, thereby significantly limiting their suitability for comprehensive cancer research.^[^ ^9^ ^]^ In addition to scientific concerns, reliance on animal models is increasingly associated with substantial ethical,^[^ ^10^ ^]^ financial,^[^ ^11^ ^]^ and regulatory burdens,^[^ ^7^, ^12^ ^]^ driving global initiatives toward more reliable, humane, and economically viable alternatives. For instance, regulatory mandates, including the National Institute of Health's (NIH's) requirement for gender‐balanced animal studies to improve rigor and reproducibility,^[^ ^13^, ^14^ ^]^ further intensify the complexity, cost, and logistical challenges of animal‐based research, catalyzing the pursuit of innovative human‐relevant experimental platforms.^[^ ^1^, ^2^, ^12^, ^15^, ^16^ ^]^

Emerging drug discovery and precision medicine will rely on human‐specific in vitro models for validation,^[^ ^17^, ^18^, ^19^ ^]^ reducing dependence on animal studies.

On the other hand, traditional in vitro systems, such as 2D cell cultures, although foundational for initial biological and pharmacological studies, inherently lack critical aspects of native tissue complexity.^[^ ^1^, ^2^, ^3^, ^4^, ^5^, ^6^, ^7^, ^8^, ^15^, ^16^, ^20^ ^]^ These simplified systems inadequately represent spatial tissue architecture, 3D cellular interactions, mechanical stimuli, and extracellular matrix (ECM) organization, markedly limiting their physiological relevance and translational potential.^[^ ^1^, ^16^, ^20^ ^]^ To address these critical shortcomings, 3D culture techniques, including spheroids and organoids,^[^ ^21^, ^22^ ^]^ have emerged, significantly enhancing experimental fidelity by incorporating elements of cellular diversity and structural complexity. Despite notable improvements, existing 3D models frequently fail to replicate the full complexity,^[^ ^23^ ^]^ diversity,^[^ ^24^ ^]^ and dynamic conditions characteristic of in vivo human tissues.^[^ ^20^ ^]^ Advanced bioengineering methods, such as microphysiological systems (MPSs)^[^ ^25^, ^26^ ^]^ and organ‐on‐a‐chip platforms,^[^ ^2^, ^27^ ^]^ have sought to overcome these limitations through sophisticated microfabrication,^[^ ^2^, ^25^ ^]^ controlled fluid dynamics,^[^ ^26^ ^]^ and mechanical stimulations.^[^ ^2^, ^6^ ^]^ Nevertheless, significant challenges persist in achieving long‐term cellular viability,^[^ ^2^, ^28^ ^]^ accurately mimicking mechanical cues,^[^ ^27^, ^28^, ^29^ ^]^ and faithfully capturing cellular heterogeneity and ECM complexity.^[^ ^2^, ^3^, ^4^, ^5^, ^7^, ^15^, ^30^, ^31^, ^32^ ^]^

Fundamentally, the recreation of tissue heterogeneity remains essential for constructing experimental models that genuinely reflect native tissue complexity, functionality, and disease progression dynamics.^[^ ^2^, ^3^, ^29^ ^]^ This includes accurately replicating the spatial arrangement and interactions among diverse cell populations and ECM components. Models with authentic cellular heterogeneity and accurate microenvironmental characteristics are particularly vital for elucidating the intricate interactions underlying pathologies such as cancer,^[^ ^2^, ^28^ ^]^ where tumor–stromal interactions,^[^ ^33^, ^34^, ^35^ ^]^ cellular diversity,^[^ ^34^, ^35^, ^36^ ^]^ and ECM dynamics play decisive roles in disease progression,^[^ ^32^, ^33^, ^36^ ^]^ therapeutic resistance,^[^ ^27^, ^36^ ^]^ and treatment efficacy.^[^ ^20^, ^30^, ^37^ ^]^

Specifically on the colon, recently, there have been some works reported, such as the integration of 2D intestinal epithelial monolayers^[^ ^20^ ^]^ and micropatterning techniques to control the molecular signal and guide the formation of the intestinal epithelium.^[^ ^38^, ^39^ ^]^ Another endeavor used intestinal stem cells organoids coupled to a MPS to generate a hybrid microchip system with luminal perfusion and medium supply.^[^ ^21^, ^29^ ^]^ Also, some researchers tried to scale up columnar crypt–villus models to seed colon epithelial cells.^[^ ^34^ ^]^ However, nearly all existing in vitro colon models inadequately represent—if at all—the inherent tissue heterogeneity and mesoscale architectural cues, significantly limiting their predictive accuracy for the in vivo human colon.^[^ ^40^ ^]^

Subsequently, precise disease modeling and drug discovery have consistently faced significant challenges due to inherent limitations of conventional preclinical models, which are not capable of replicating the complex,^[^ ^23^ ^]^ and dynamic microenvironment characteristic of human tissues.^[^ ^31^, ^34^, ^41^ ^]^

Addressing these critical gaps, we introduce an innovative, state‐of‐the‐art, 3D in vivo mimicking human colon model (3D‐IVM‐HC model) that more precisely replicates the complex microenvironment of the human colon with unparalleled fidelity. The colon was selected as our model due to its critical roles in digestion, protein synthesis, nutrient absorption, the symbiotic relation with the microbiota, and its high susceptibility to diseases such as colon cancer, a leading global health concern.^[^ ^42^ ^]^ Using computed‐tomography (CT)‐derived anatomical data, we precisely engineered a scaled‐down replica that accurately incorporates essential structural features, including luminal curvature, multilayered cellular organization, and the spontaneous formation of crypt‐like domains. This biomimetic 3D‐IVM‐HC model meticulously captures intricate mucosal architecture, heterogeneous cellular composition, and distinct luminal topography, surpassing the physiological accuracy of existing experimental systems. In addition to structural fidelity, the model reproduces dynamic conditions through luminal curvature‐driven epithelial polarization, spontaneous crypt formation, and functional stromal–epithelial interactions, capturing essential features of in vivo tissue dynamics.

Our heterogeneous 3D‐IVM‐HC model promotes the spontaneous formation of crypt‐like folds resulting from epithelial proliferation and aggregation on curved surfaces. While these structures resemble aspects of native crypt morphology, they do not yet represent fully functional crypts, with median fold heights (≈65 µm) confirmed via immunofluorescence microscopy.

This intricate architectural arrangement promotes robust cell‐to‐cell interactions, significantly increasing cell density fourfold relative to conventional 2D cultures, thus enhancing physiological relevance and barrier functionality. Moreover, immediate postprinting cell viability exceeded 90%, substantially outperforming traditional methods^[^ ^15^, ^43^, ^44^ ^]^ and underscoring the model's viability and practical advantage.

Our 3D‐IVM‐HC model demonstrated superior physiological relevance compared to traditional culture systems. Specifically, transepithelial electrical resistance (TEER) assays confirmed markedly enhanced epithelial barrier integrity relative to conventional 2D and 2.5D coculture systems. Furthermore, functional drug‐response assays revealed significantly increased chemoresistance (higher IC_50_ values^[^ ^39^, ^45^ ^]^) against 5‐fluorouracil compared to traditional 2D Transwell cultures, reflecting a more realistic, clinically relevant response. Our 3D‐IVM‐HC model also better captures the complexity of cancer–stroma interactions, providing a more precise platform for investigating tumor biology and therapeutic responses with better accuracy and predictive power. Additionally, our 3D‐IVM‐HC model addresses substantial practical and ethical issues inherent in animal‐based research.^[^ ^10^ ^]^ Animal models are notoriously resource‐intensive, demanding extensive financial investments, prolonged timelines, and complex regulatory compliance.^[^ ^12^, ^14^, ^46^ ^]^ For instance, they require financial resources that often total multiple millions of dollars over 4–5 years just for routine cancer studies.^[^ ^30^, ^47^ ^]^ By utilizing our human‐cell‐based, animal‐free approach, the 3D‐IVM‐HC model substantially reduces experimental costs (by ≈70–80%, required for animal procurement, housing, and regulatory compliance)^[^ ^9^, ^14^, ^46^ ^]^ and streamlines timelines, thus facilitating rapid, cost‐effective, more ethical, and scalable translational studies. Importantly, eliminating interspecies variability in our 3D‐IVM‐HC model is expected to enhance clinical translatability, providing an accelerated and ethically responsible pathway for preclinical research.

Collectively, our first‐of‐its‐kind biomimetic 3D‐IVM‐HC colon model can potentially establish an innovative benchmark in colorectal cancer research by bridging critical gaps in preclinical modeling and drug evaluation. By more precisely replicating the structural complexity, cellular diversity, and intricate cancer–stroma interactions of native human colon tissue, this innovative non‐animal model can potentially advance mechanistic understanding, enhance predictive accuracy in therapeutic efficacy assessments, and accelerate high‐throughput precision drug discovery. Ultimately, our integrated approach holds transformative potential for personalized medicine, promising significant translational impacts and improved clinical outcomes for colorectal cancer patients and beyond.

## 2 Results

### 2.1 Design and Fabrication of Colon Structure

**Figure 1:**
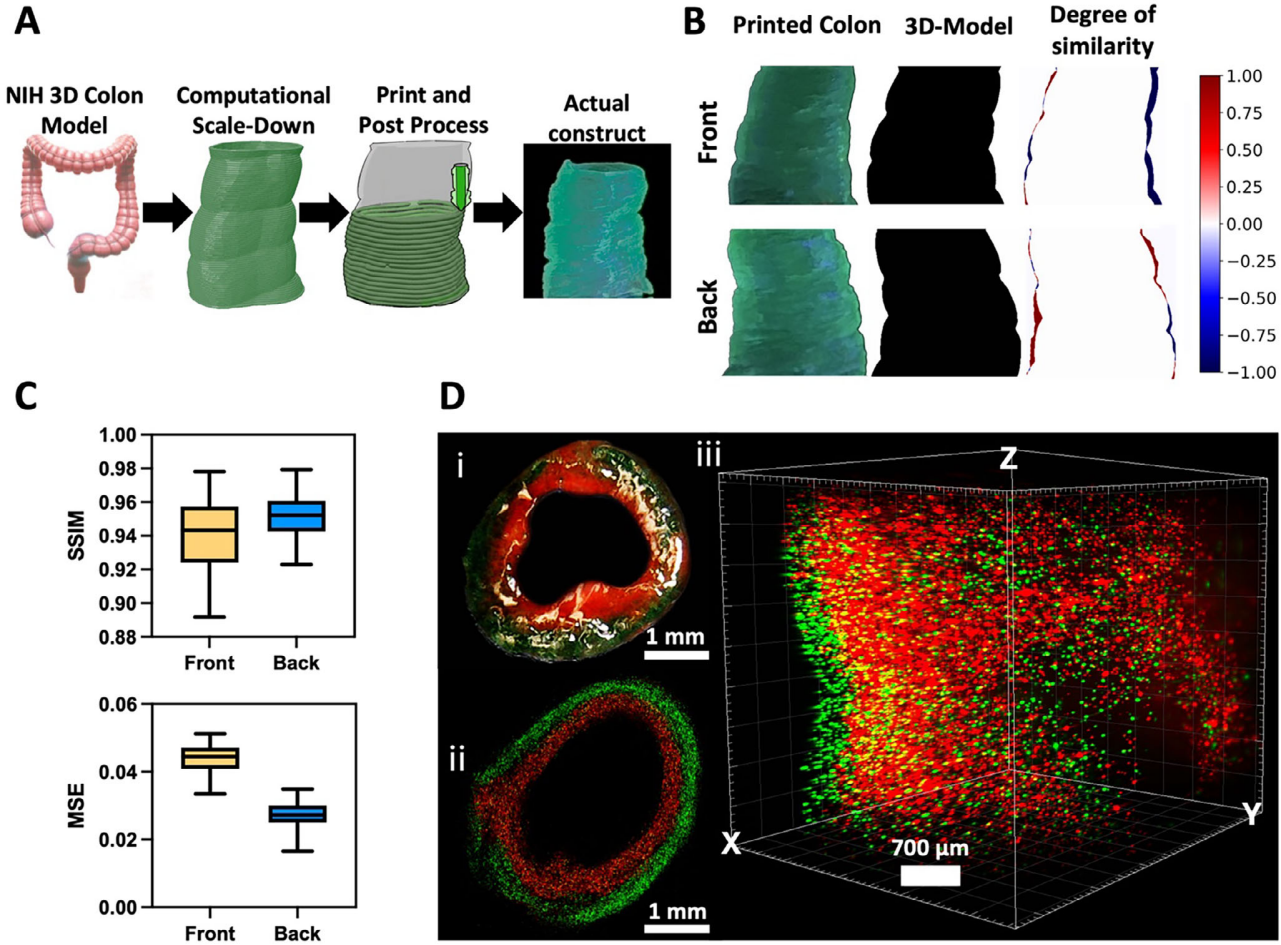
Design and biofabrication of the 3D‐IVM‐HC model. A) Segmentation of a human colon 3D CT scan, computational processing of the 3D CT scan, biofabrication process using the FRESH method, and the actual 3D‐IVM‐HC construct. B) Fidelity comparison of the 3D‐IVM‐HC model and 3D CT scan. The color map indicates the degree of similarity, with blue representing negative differences and red indicating positive differences. C) Box and whiskers graph of the resolution assay's comparison metrics, structural similarity index, and mean square error. D) Optical characterization of the printed colon. i) Cross‐section photograph of the double‐layered printed 3D‐IVM‐HC model with fluorescent particles. ii) Fluorescent microscopy image of the cross‐section. iii) Light‐sheet microscopy image reconstruction of the double‐layered printed 3D‐IVM‐HC model. Fidelity analysis (panels B, C) was performed on orthogonal 2D images after removal from the support bath, processed in ImageJ, and compared to the STL template. SSIM quantified structural similarity (luminance, contrast, and spatial structure), while MSE measured average pixel‐wise error.

The colon, constituting the initial segment of the large intestine, is a muscular tube ≈1.5 m (5 feet) long and 5 cm (2 in.) in diameter, essential for water and electrolyte absorption and feces elimination.^[^ ^48^ ^]^ Structurally, all segments of the human intestine, including the colon, are composed of four distinct layers: the intestinal epithelium, subepithelium, muscle layer, and serosa.^[^ ^48^, ^49^ ^]^ The epithelium is intricately connected to a mesenchymal stroma network, consisting of fibroblasts, myofibroblasts, endothelial cells, pericytes, immune cells, and neural cells.^[^ ^49^ ^]^ In this study, to fabricate our 3D‐IVM‐HC, we utilized a 3D colon model derived from human colon CT data, provided by the NIH for research purposes.^[^ ^50^ ^]^ As illustrated in **Figure** **1**A, the CT model was scaled down to approximate dimensions of 10 mm in length and 5 mm in diameter, while preserving the critical 3D architecture and curvature of the native intestine—features essential for maintaining physiologically relevant cell behavior and functionality. This downscaled model features an inner hollow lumen that facilitates the in vitro reconstruction of a functional 3D intestinal epithelial interface. Concurrently, the outer layer is designed to support fibroblasts, serving as stromal cells that promote intestinal epithelial differentiation and functionality.^[^ ^34^, ^49^ ^]^

To accurately replicate the colon's structure in our 3D‐IVM‐HC model, we implemented the FRESH 3D bioprinting technique to enhance printing stability and precision. FRESH enables the use of low‐viscosity hydrogels like gelatin methacrylate (GelMA) by providing structural support during printing, with alginate acting as a thickener to further stabilize the hydrogel matrix.^[^ ^51^ ^]^ This combination overcomes challenges related to deformation and insufficient mechanical stability, allowing for the precise creation of complex and stable structures that closely mimic the mechanics and architecture of colon tissue.

Building on our 3D bioprinting experties, we studied four bioink formulations: 5% and 7.5% GelMA, a blend of 7.5% GelMA with 0.25% alginate, and 7.5% GelMA with 0.5% alginate, aiming to achieve optimal printability, structural integrity, mechanical strength, and cell growth in our 3D‐IVM‐HC model. All bioinks exhibited shear‐thinning properties essential for 3D bioprinting,^[^ ^52^, ^53^, ^54^ ^]^ with increased GelMA concentration and alginate addition enhancing viscosity, demonstrated through rheological tests (Note S1 and Figure S1, Supporting Information) However, for superior structural integrity, particularly in freestanding models, viscosity alone is insufficient. Notably, the 7.5% GelMA with 0.5% alginate bioink excelled in maintaining its shape within the support bath, attributed to synergistic cross‐linking.^[^ ^54^ ^]^ Furthermore, we showed that the increase in GelMA concentration and the incorporation of alginate would increase the viscosity of the bioink. The GelMA, activated by lithium phenyl‐2, 4, 6‐trimethylbenzoylphosphinate (LAP) (a biocompatible photoinitiator), undergoes UV photo‐cross‐linking, forming covalent bonds. Alginate's ionic cross‐linking, triggered by calcium ions in the support bath, further stabilizes the structure. This dual‐cross‐linking mechanism, utilizing a biocompatible bioink, significantly improves postprinting shape retention.^[^ ^54^ ^]^ Our optimized protocol leverages both composition and cross‐linking strategies for tailored applications.

To evaluate the performance of generating complex, heterogeneous colon tissues that incorporate multiple cell types or bioinks, we developed a double‐layer 3D bioprinting approach tailored for our 3D‐IVM‐HC model fabrication. This method involved using two distinct bioink compositions to replicate different functional layers of the colon's structure. The CT scan was sliced into 100 layers in the *Z*‐direction; we started printing with the outer layer before adding the inner layer. Following the printing process, we used UV light (405 nm) for 1 min to photo‐cross‐link the structure, ensuring its stability. Finally, the construct was placed in an incubator for 30–40 min. This step allowed the support bath to fully dissolve, freeing the 3D bioprinted colon structure. We've documented the result of this process, providing a side view and top view of the actual construct in Figure 1A, with further protocol details available in Movie S1 (Supporting Information). Furthermore, we sought to quantitatively assess the fidelity of 3D‐bioprinted constructs in comparison to their corresponding 3D CT model using a series of image comparison metrics, as shown in Figure 1B.

Moreover, we aimed to quantitatively evaluate the fidelity of the 3D‐IVM‐HC‐printed constructs against their corresponding 3D CT models using a set of image comparison metrics. Using image processing techniques, including conversion to HSV color space and color‐specific thresholding, we generated binary masks to facilitate a precise analysis of the constructs against their backgrounds. Additionally, by comparing randomly selected 50 perimeter points, we ensured accurate differentiation and assessment. This preparation allowed us to apply the structural similarity index (SSIM) and mean squared error (MSE) metrics to evaluate the visual and structural fidelity of the printed colon to its 3D model counterparts,^[^ ^52^, ^55^ ^]^ as illustrated in Figure 1C. Our findings, highlighted by high SSIM values (0.9401 ± 0.074 and 0.9521 ± 0.066 for back and front views, respectively) and low MSE values (0.0447 ± 0.003 and 0.0266 ± 0.001 for back and front views, respectively), demonstrated a strong alignment and minimal pixel‐wise discrepancies between the 3D‐IVM‐HC‐printed construct and the 3D CT models, underscoring the precision of our 3D bioprinting process. Fidelity was quantified by comparing orthogonal images of the printed constructs to the CT‐derived STL model using the SSIM and MSE. SSIM measures image similarity in terms of structure, luminance, and contrast, while MSE quantifies the average squared difference in pixel intensity. While OCT‐based in situ imaging approaches, such as those reported by Tashman et al.,^[^ ^56^ ^]^ allow full volumetric fidelity assessment inside the support bath, our analysis was performed after release from the support bath, imaging the constructs in air. This postrelease approach reflects the geometry in the state actually used for biological culture, though minor deformation from gravity or drying may occur. Orthogonal 2D images were captured with a digital camera, processed in ImageJ (HSV thresholding, binary mask generation, and perimeter point extraction), and compared with the CT‐derived STL template. Although this method does not provide a complete volumetric reconstruction, it yields robust SSIM and MSE values, demonstrating that the released constructs maintain high fidelity to the intended geometry.

By incorporating green and red fluorescent polymer beads into the ink, we studied the resolution of the 3D‐IVM‐HC structures. Figure 1D presents a photograph and fluorescent microscopy image of a cross‐section and the light‐sheet reconstruction of the printed colon with fluorescent beads. The cross‐section reveals a double‐layered structure with intimate integration between the layers, as further confirmed by light‐sheet microscopy reconstruction, which illustrates the distribution of fluorescent particles across the two layers in the 3D and the replication of the curve architecture of the colon.

#### 2.1.1 Characterization of the Hydrogels

**Figure 2:**
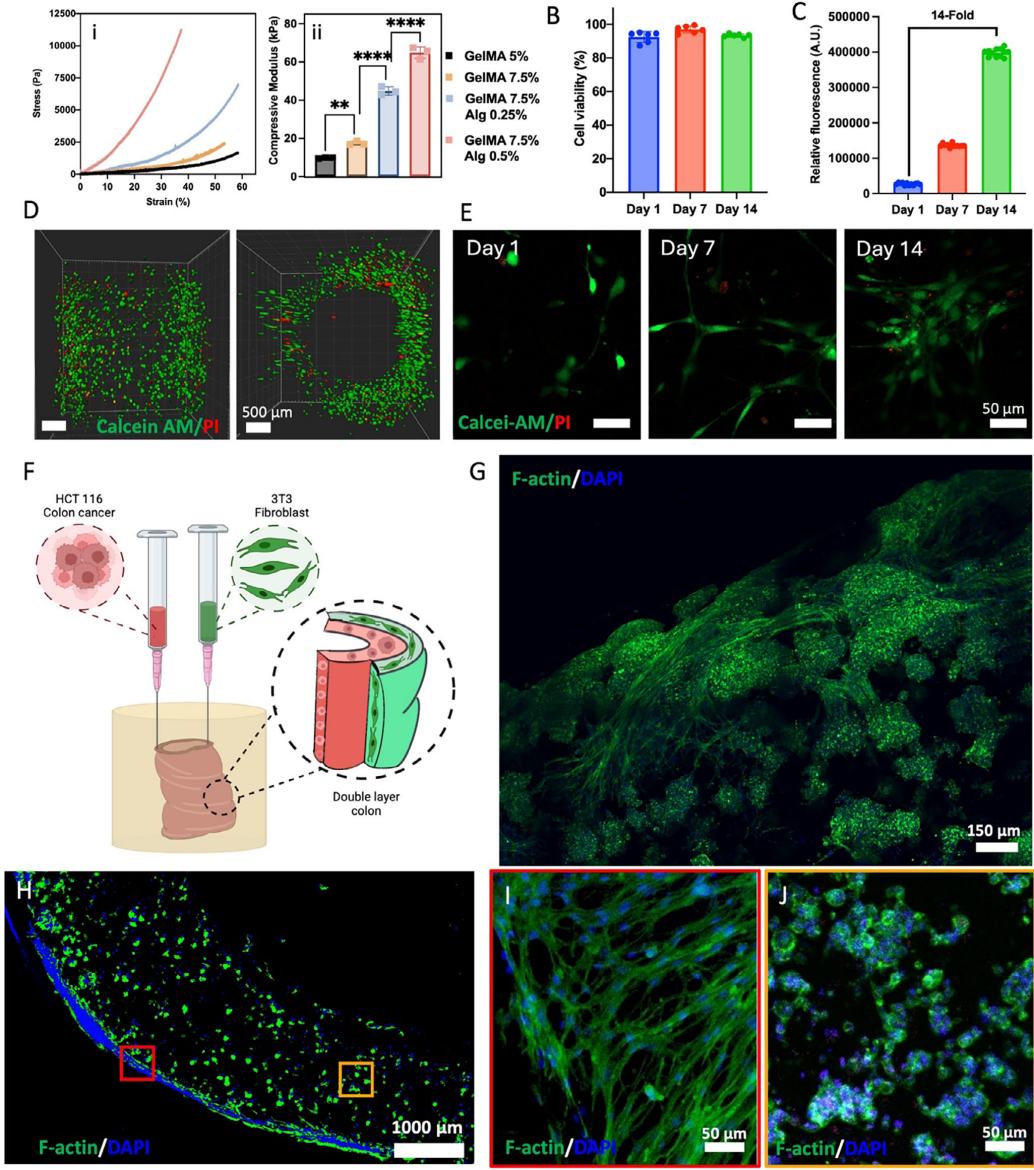
Characterization of the bioprinted 3D‐IVM‐HC constructs. A) Mechanical characterization of the hydrogels. i) Stress–strain curves of GelMA (gelatin methacrylate) at 5% and 7.5%, and GelMA 7.5% combined with 0.25% or 0.5% alginate. ii) Compressive modulus of the hydrogels. B) From this panel onward, experiments were performed using the optimized GelMA 7.5%–alginate 0.5% formulation. Cell viability of bioprinted constructs at days 1, 7, and 14, assessed by Calcein‐AM (live, green) and propidium iodide (PI, dead, red) staining. C) Normalized metabolic activity (resazurin assay) at days 1, 7, and 14. D) Whole‐mount 3D light‐sheet microscopy image of the bioprinted 3D‐IVM‐HC model stained with Calcein‐AM/PI on day 1, with the lateral and transversal views. E) Calcein‐AM/PI staining for cell viability. F) Scheme of the 3D bioprinting procedure of the two‐layer colon containing 3T3 fibroblast and HCT116 human colorectal carcinoma cell line. G) Fibroblast and carcinoma cell proliferation within the 3D bioprinted colon on day 21, visualized using F‐actin/DAPI staining. H) Cross‐sectional visualization of cell distribution on the surface and within 3D‐IVM‐HC constructs stained with F‐actin/DAPI on day 14. The squares indicate two distinct regions: the red square for the external fibroblast layer, while the yellow square marks the internal layer of cancer cells. I) Magnified view of the external 3T3 fibroblast layer corresponding to the red square. J) Magnified view of the HCT116 cancer cells growing in the internal layer corresponding to the yellow. Error bars represent the standard error of the mean; asterisks indicate significance levels of *p* < 0.01 (**) and *p* < 0.0001 (****), with *n* ≥ 5.

To determine if our hydrogel formulations mimic the mechanical properties of fibrotic soft tissues, we conducted compression tests on four bioinks: GelMA at 5% and 7.5%, and GelMA 7.5% mixed with 0.25% and 0.5% alginate, respectively, using an Instron system (**Figure** **2**A‐i,ii). Results showed a progressive increase in the Young's modulus (*E*) with alginate addition, from 9.8 ± 0.3 kPa for GelMA 5%, and 15.8 ± 0.2 kPa for GelMA 7.5%, to 43 ± 2.4 and 65 ± 8.5 kPa for mixtures with 0.25% and 0.5% alginate, respectively (Figure 2A‐ii). Bulk compression of molded disks, cross‐linked under the same dual protocol as the bioprinted constructs, provides standard and reproducible bulk mechanical data. Advanced techniques such as micro‐ or nanoindentation may complement these results in future work. These results align with reported colon tissue mechanics, which can span ≈1–100 kPa. Although some references document lower moduli for healthy tissue,^[^ ^33^, ^36^ ^]^ the dual‐cross‐linking approach required for robust 3D bioprinting yields a final modulus of about 65 kPa. By positioning our construct near the mid‐range of this broader physiological spectrum, we achieve superior structural fidelity and mechanical resilience—key factors for preserving crypt‐like architecture under cyclical forces. This, in turn, fosters a stable environment for realistic cell behavior and tissue functionality in vitro. The working formulation of GelMA 7.5%–alginate 0.5% was selected based on multiple criteria. Metabolic activity and fibroblast elongation were considered for this decision, in addition to printability, fidelity, and stability. Fibroblasts cultured in this matrix exhibited more elongated morphologies, consistent with enhanced cell–matrix interactions, and the formulation maintained comparable or higher metabolic activity compared to softer candidates. Although its modulus (65 ± 8.5 kPa) is higher than the reported physiological range for colon tissue (0.6–7.3 kPa), softer formulations (5% or 7.5% GelMA without alginate) could not preserve structural integrity in multilayered constructs and frequently collapsed during printing. For this reason, a slightly stiffer formulation was prioritized to achieve anatomical fidelity and reproducibility. We acknowledge this stiffness mismatch as a limitation and note future directions to mitigate it, including the incorporation of softening agents, dynamically degradable materials, or ECM‐mimicking bioinks.

#### 2.1.2 Interaction between Cells and Matrix

We studied various GelMA and alginate volume ratios to optimize 3T3 fibroblast adhesion, proliferation, and elongation. In this research, 3T3 fibroblasts were selected as foundational stromal cells to recreate the native environment of the colon, providing essential support for cellular interactions within our 3D‐IVM‐HC model. Embedding 3T3 fibroblasts in differing hydrogel mixes, we assessed cell growth by staining the cell cytoskeleton with phalloidin, which binds to F‐Actin. The findings revealed that higher GelMA concentrations coupled with lower alginate percentages favored the formation of a more dispersed and elongated fibroblast network, as opposed to dense cell aggregations (Note S2 and Figure S2, Supporting Information). Notably, the GelMA 7.5% and alginate 0.5% composition emerged as particularly conducive for colon tissue engineering, striking a balance between printability, structural integrity, and the biomechanical environment necessary for cell proliferation and tissue development. In 3D bioprinting, especially for complex structures, three critical considerations are the bioink's cytocompatibility, the cross‐linking strategy, and the shear forces applied to cells during the extrusion process.^[^ ^57^ ^]^ To assess these factors, we monitored cell viability and proliferation within our 3D‐IVM‐HC constructs over 14 days postprinting via live–dead staining and resazurin reduction as metabolic activity assay, presented in Figure 2B–E. The bar plot (Figure 2B) shows that 3T3 fibroblast viability within the constructs was over 90% on the first day after printing, increasing to more than 95% by the end of the two‐week culture period. Metabolic activity, measured via the resazurin reduction assay (Figure 2C), revealed a fivefold increase by day 7 and a 14‐fold increase by day 14. Additionally, light‐sheet microscopy reconstruction and confocal imaging provided a clear visualization of live cells distributed throughout the 3D structure on day 1, further confirming the high viability of 3T3 fibroblasts (Figure 2D). This indicates that the shear forces encountered during extrusion, subsequent embedding in a support bath, and UV light exposure did not adversely affect cell viability, as further evidenced by Figure S3 (Supporting Information) and described in Note S3 (Supporting Information).

To assess the efficacy of our hydrogel matrix in supporting complex tissue environments, we developed a bilayered construct incorporating multiple cell types (Figure 2F). In alignment with our objective of engineering a functional 3D‐IVM‐HC model for tumor formation and therapeutic evaluation, this phase of our study examined the matrix's capacity to sustain a heterogeneous tissue‐like environment. Specifically, we incorporated 3T3 fibroblasts as a stromal component and introduced HCT116 colon carcinoma cells to simulate a tumor component^[^ ^35^ ^]^ (Note S4 and Figure S4, Supporting Information). We selected HCT116 because it is one of the most widely studied human colorectal adenocarcinoma lines, featuring a well‐characterized genetic profile and aggressive growth behavior—attributes that make it a robust choice for investigating cancer–stroma interactions and therapeutic responses.^[^ ^58^ ^]^ This structural design enabled us to rigorously evaluate the matrix's capability to facilitate cellular interactions and support the physiological architecture of a colon tissue model, thereby setting the stage for future investigations into tumor growth dynamics and drug response within a controlled in vitro model. To this end, this 3D‐IVM‐HC construct was produced following a previously defined bioprinting protocol and was cultured for a duration of 21 days. Initial cell viability for both 3T3 and HCT116 cells exceeded 90% at day 1 postprinting. (Note S5 and Figure S5, Supporting Information). Notably, within the hydrogel matrix (internal layer), the HCT116 cells, known for their exacerbated proliferation, aggregated into spheroids and showed significant growth throughout the culture period.^[^ ^37^ ^]^ By day 21, these spheroids reached an average diameter of 100 µm, as detailed in Figure 2G and Note S4 and Figure S4 (Supporting Information). HCT116 cells' rapid proliferation and spheroid formation within the lumen confirmed that the hydrogel matrix could support bilayer organization with distinct stromal and epithelial‐like compartments, thereby validating the design prior to fabricating the final 3D‐IVM‐HC with Caco‐2 and 3T3 cells.

Additionally, the junctional integrity of the spheroids was evaluated using Zonula occludens‐1 (ZO‐1) staining on day 21. The confocal microscopy image shown in Notes S4 and S6 and Figures S4 and S6 (Supporting Information) illustrated well‐organized tight junctions on both the surface and within the interior of the spheroids.

The distribution of cells is also observable in Figure 2H, which shows a section of the bioprinted colon stained on day 14 with phalloidin and DAPI to visualize the cytoskeleton of fibroblasts and cancer cells. The image reveals a distribution of cells throughout the structure, with a higher concentration at the surface of the 3D‐IVM‐HC model. The migratory behavior of the fibroblasts was evident as they moved toward the periphery of the construct and elongated, as demonstrated by immunostaining for DAPI and F‐actin in the 3T3‐laden bioprinted colon construct at day 14. Zoomed‐in views of specific areas are presented in Figure 2I for fibroblasts and Figure 2J for cancer cells. In these images, the 3T3 fibroblasts on the construct's surface can be seen elongating and forming an interconnected network, contributing to the construct's mimicry of native colon tissue architecture.^[^ ^48^ ^]^ Simultaneously, HCT116 cells aggregate into spheroids within the internal layer of the 3D‐IVM‐HC model. To preserve the construct's morphology for imaging, we embedded the hydrogel samples in 1% agarose gel and sectioned them into 1 mm slices (Note S7 and Figure S7, Supporting Information). The detailed procedure is provided in the Experimental Section. Our 3D‐IVM‐HC model demonstrated its efficiency in supporting complex tissue environments and effectively recapitulating the native mechanical, structural, and physiological properties of colon tissue, providing a realistic tumor–stroma microenvironment for colorectal research.^[^ ^3^, ^34^, ^35^ ^]^ Furthermore, it serves as a viable ethical alternative to animal testing, reducing reliance on in vivo models.^[^ ^9^, ^12^ ^]^

#### 2.1.3 Development of a Functional 3D In Vivo Mimicking Model of the Colon

**Figure 3:**
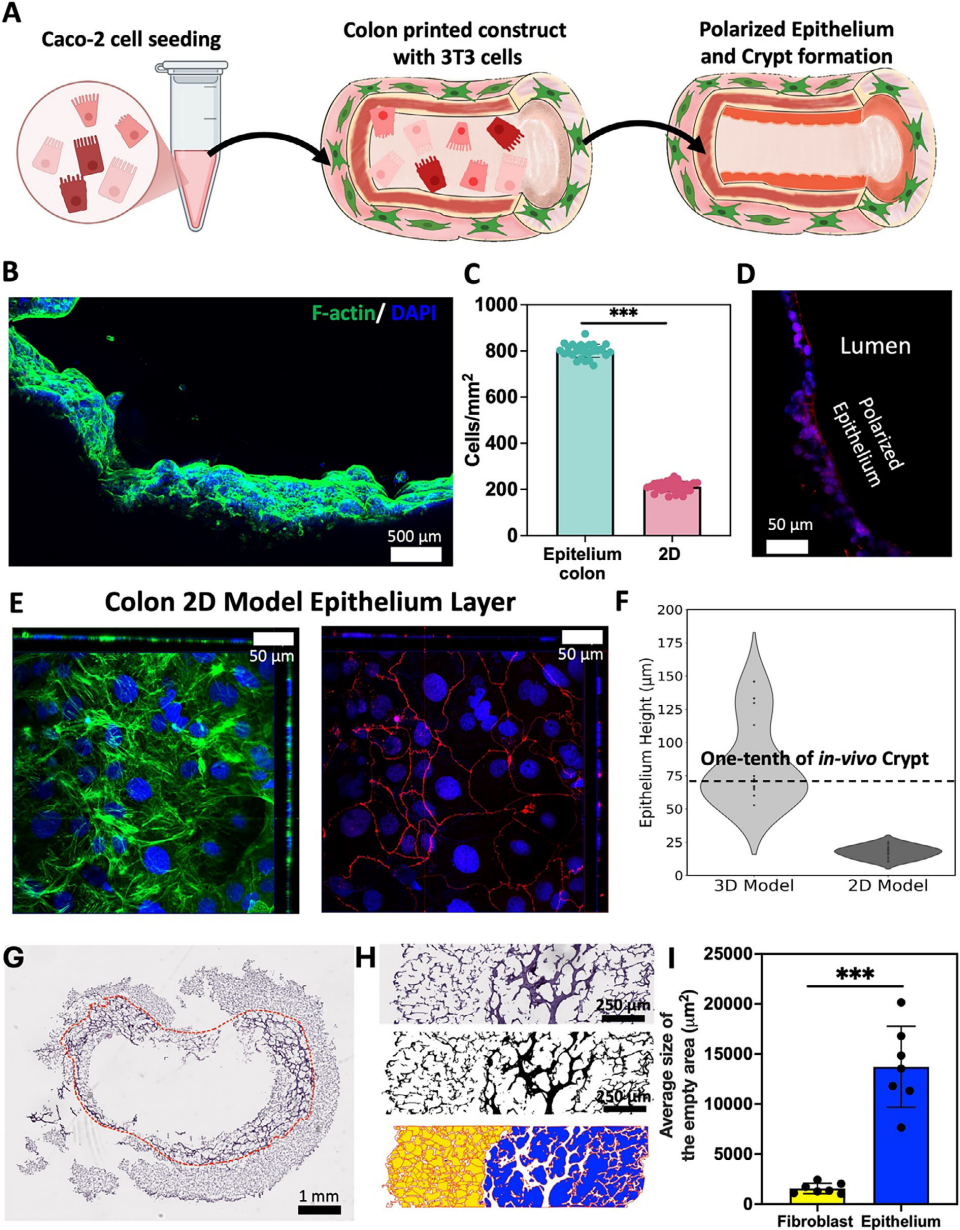
Characterization and comparison of 3D‐IVM‐HC epithelium and the 2D Transwell model. A) Schematic of the 3D‐IVM‐HC construct with Caco‐2 cell seeding, showing progression to a polarized epithelium with crypt formation. B) Lateral view immunofluorescent depiction of Caco‐2‐cell‐mediated epithelial coverage at day 14, highlighting the compact, confluent barrier formation as evidenced by DAPI and F‐actin staining. C) Comparison of cell density between the Caco‐2 cells in the 3D‐IVM‐HC model and the 2D culture. D) A fluorescent microscopy image of the polarized and differentiated epithelium expressing ZO‐1 (in red) indicates the formation of tight junctions between cells, signifying the establishment of a polarized and differentiated epithelium. E) The 2D Transwell model with Caco‐2 cells forming a tight, continuous monolayer, exhibiting F‐actin (in green), cell nuclei (in blue), and ZO‐1 (in red). F) A violin plot comparing the distribution of epithelium heights in the 3D‐IVM‐HC model to the epithelium height in the 2D model, demonstrating the morphological superiority of the 3D‐IVM‐HC model in mimicking in vivo‐like crypt structures, with heights approximately one‐tenth that of native intestinal crypts, as measured by immunofluorescence. G) Transversal H&E staining of bioprinted 3D‐IVM‐HC constructs with a dashed line separating the stromal (outer) and epithelial (inner) layers. Larger voids in the epithelial region reflect epithelial cell footprints and crypt‐like organization, while smaller, denser voids in the stromal region reflect fibroblast networks. H) Image processing and binarization of the fibroblast and epithelial layers. I) Quantification of the average void area in stromal versus epithelial regions, confirming bilayer distribution.

The development of a 3D‐IVM‐HC model, which accurately replicates the physiological architecture and functionality of the native colon, requires precise reconstruction of the epithelial and subepithelial layers.^[^ ^59^, ^60^ ^]^ The intestinal tract is characterized by a monolayer of columnar epithelium, predominantly consisting of absorptive enterocytes.^[^ ^59^ ^]^ These enterocytes create a continuous, protective barrier of tightly packed cells with a minimal intercellular matrix, essential for the physiological integrity of the gastrointestinal tract.^[^ ^49^, ^61^ ^]^ To comprehensively mimic the functionality of colon models in vitro, the incorporation of the epithelial and subepithelial layers is imperative. Nonetheless, the resolution limitations of 3D bioprinting technology pose a challenge in achieving the precision required for the single‐cell layer fabrication. As an initial step, we fabricated the 3D‐IVM‐HC model utilizing a 3T3‐cell‐laden hydrogel. Within our engineered 3D scaffolds, a central hollow lumen facilitates the establishment of a functional epithelial interface in vitro. On the seventh day postprinting, Caco‐2 cells, which serve as a model for human colorectal epithelial enterocytes, were seeded into the hollow lumen of the 3D‐IVM‐HC model. This process was aimed at forming an epithelial barrier, as illustrated in **Figure** **3**A. Over a period of 14 days, these cells uniformly and continuously coated the structure, culminating in the formation of a robust epithelial barrier contiguous with the lumen of the 3D‐IVM‐HC model. Immunofluorescence imaging at day 14 postcultivation demonstrates the epithelial confluence achieved by Caco‐2 cell coverage, as evidenced by DAPI and F‐actin staining. This illustrates cellular proliferation and the formation of a dense, continuous barrier layer. One major difference between 2D and 3D tissue models is the number of cells contained within them, which directly influences the functional relevance of the engineered system.^[^ ^31^, ^62^ ^]^ Traditional 2D models typically achieve a cell density of only a few hundred cells per mm^2^, whereas 3D models accommodate a significantly higher number of cells by providing a more physiologically relevant microenvironment.^[^ ^31^ ^]^ The increased cell density in 3D models is crucial for mimicking the cellular interactions, tissue architecture, and barrier properties observed in native colon tissue. In this context, we utilized fluorescent imaging (Figure 3B) to compare the cell density between a control conventional Caco‐2 monolayer and the Caco‐2 cells seeded within the lumen of the 3D‐IVM‐HC model. The imaging analysis revealed that the 3D‐IVM‐HC model exhibited a fourfold higher cellular density compared to the 2D monolayer (Figure 3C), with cells uniformly distributed along the lumen. This difference was statistically significant (*p* < 0.001) and proved the enhanced capacity of the 3D‐IVM‐HC model to support higher cell numbers. The increased cellular density in the 3D‐IVM‐HC model may strengthen the structural integrity of the engineered tissue and improve the cell‐to‐cell communication, polarization, and barrier function.

Moreover, immunofluorescence images depicted the development of crypt‐like structures toward the lumen that resemble the morphology found in human colon tissue. A zoomed‐in view of these structures, as shown in Note S8 and Figure S8 (Supporting Information), reveals crypt‐like structures extending toward the hollow lumen, with cell nuclei stained in blue and actin filaments in green. This phenomenon is explained by the 3D architecture of the 3D‐IVM‐HC model and its luminal curvatures influencing epithelial cell behavior to mature, polarize, and differentiate to form crypt‐like structures, as previous studies have shown that curved surfaces contribute to apical‐basal polarization that increases intestinal epithelial barrier functions.^[^ ^28^, ^63^, ^64^ ^]^ In contrast to Abdollahi et al.,^[^^]^ who employed micropatterned villus–crypt biomimetic substrates to enhance epithelial differentiation at the microscale, our model leverages macroscale luminal curvature that parallels the tubular anatomy of the colon. Such curvature not only reinforces polarization but also promotes epithelial maturation, tissue self‐organization, and spatial compartmentalization through curvature‐dependent mechanical and biochemical cues.^[^ ^28^, ^63^ ^]^ This distinction highlights the complementary roles of microscale topographical patterning and macroscale curvature in shaping intestinal tissue architecture and function. In addition, we acknowledge that the groove‐like patterns visible between bioprinted layers, which arise from limited *Z*‐axis resolution, may themselves act as unintended microtopographical cues. Such features could potentially reinforce epithelial polarization and folding behavior, similar to the effects of engineered biomimetic substrates reported by Abdollahi et al.^[^ ^65^ ^]^ These emergent microstructures highlight the dual influence of intentional design (luminal curvature) and unintentional bioprinting artifacts in shaping epithelial growth and organization, warranting further investigation.

The polarized epithelium, typical of mature enterocytes, indicates that our 3D‐IVM‐HC model architecture provided a suitable 3D niche for intestinal epithelial cells to attach and differentiate^[^ ^21^, ^64^ ^]^ (Figure 3D). To confirm that this epithelium also establishes functional barrier properties, we assessed the tight junction protein ZO‐1, a key marker of fully differentiated intestinal epithelial cells. Robust ZO‐1 immunostaining was observed throughout the 3D‐IVM‐HC lumen (Figure 3D; Figure S8, Supporting Information), confirming that the epithelial cells form tight junctions essential for barrier integrity.

In intestinal tissue engineering, using polarized Caco‐2 epithelial cells cultured on planar Transwell inserts is a well‐established method for studying epithelial interactions with pathogens. This approach provides a physiologically accurate model for investigating the mechanisms underlying intestinal barrier function and pathogen invasion.^[^ ^66^ ^]^ This study employs a superior approach compared to other models by incorporating fibroblasts beneath the Transwell inserts to simulate the stromal layer, while Caco‐2 cells are cultured on the upper surface of the membrane to constitute the epithelial component. This configuration more accurately replicates the native cellular microenvironment of the human intestine, enabling enhanced investigation of epithelial–stromal interactions. Following a 14 days maturation period, we employed immunofluorescence microscopy to investigate the expression of the intestinal epithelial differentiation marker, ZO‐1, cell nuclei, and actin structures.

Figure 3E illustrates that the Caco‐2 cells within this Transwell arrangement developed tight junction patterns indicative of monolayer integrity.^[^ ^67^ ^]^ To further evaluate cellular morphology, we generated Z‐stacks, allowing for the inspection of epithelium morphology between 2D and 3D growth conditions. The Caco‐2 cells in Transwell consistently exhibited a flat monolayer formation of the epithelium layer. The quantitative assessment of epithelium height in both Transwell and the 3D‐IVM‐HC model, showcased in Figure 3F, revealed an epithelium height of around 20 µm for the 2D Transwell format, and a range from 20 to 180 µm in the 3D configuration. This scale is approximately one‐tenth of the native intestinal internal microstructure, which has been measured at 400–1000 µm in height in situ.^[^ ^68^ ^]^ Our 3D‐IVM‐HC model's crypt‐like structures’ height distribution is summarized in a violin plot, highlighting that the majority of crypt‐like structures’ heights cluster around the median of 65 µm. This median approximates one‐tenth of the in vivo median crypt‐like structures’ height, with a span ranging from 20 to 180 µm.^[^ ^68^, ^69^ ^]^ The measurements for crypt‐like structures’ height were conducted using immunofluorescent imaging, with the baseline for measurement delineated in the images (Figure S8, Supporting Information).

A commonly noted shortcoming of current intestine‐on‐chip models is their lack of physiological replication of the diverse intestinal epithelial subtypes along the microarchitecture structure.^[^ ^26^ ^]^ By contrast, our 3D‐IVM‐HC model exhibits a morphology that more closely resembles the in vivo colon tissue architecture, surpassing traditional 2D Caco‐2 cell monocultures on Transwell systems. These conventional models fail to develop crypt‐like structures, displaying only apical actin filament expression after two weeks of culture. Our enhanced 3D‐IVM‐HC model, therefore, presents a more physiologically relevant in vitro system for the examination of epithelial integrity and function. A defining innovation of our 3D‐IVM‐HC model lies in its sophisticated biomimetic architecture, featuring the spontaneous formation of crypt‐like domains with accurate 3D luminal curvature and intricate mucosal architecture.^[^ ^22^, ^27^, ^70^ ^]^ This 3D structural design meticulously replicates the native layered cellular heterogeneity, including a distinct outer stromal layer populated by uniformly dispersed fibroblasts embedded in a mechanically robust extracellular matrix and an inner epithelial layer comprising aggregated Caco‐2 cells arranged into crypt‐like domains.^[^ ^34^, ^35^, ^55^, ^64^ ^]^ Such a configuration ensures localized mechanical and biological heterogeneity, closely resembling the native colon microenvironment, essential for realistic epithelial–stromal interactions.^[^ ^36^, ^71^ ^]^

To further examine the layered architecture of our 3D construct, we performed hematoxylin and eosin (H&E) staining (Figure 3G). Although H&E routinely visualizes cells in native tissues, the harsher processing steps (e.g., multiple dehydration and clearing stages) often cause cell loss in hydrogel‐based bioengineered constructs.^[^ ^34^, ^72^ ^]^ Consequently, what remains here is predominantly the matrix and empty voids where cells had been. Notably, the inner (epithelial) region contains much larger voids—consistent with earlier observations of crypt‐like cell aggregation—while the outer (stromal) region appears denser. We segmented the histological image into 500 × 500 µm subregions to quantify these differences and binarized each segment to distinguish the matrix from voids. The epithelial side exhibited approximately sixfold more void area, and larger cavities were left behind by the once closely packed enterocytes, forming crypt‐like structures. By contrast, the stromal layer retained a more uniform matrix distribution and fewer voids, reflecting tighter fibroblast‐laden networks that provide mechanical support. Taken together, these H&E findings corroborate the formation of distinct epithelial and stromal layers, illustrating how our 3D‐bioprinting strategy replicates the colon's multilayer organization. While the cells themselves are not visible in this staining due to their loss during histological preparation, the morphological “footprints” they leave behind validate the construct's potential to mimic native colon complexity. While conventional staining methods (e.g., H&E staining) posed challenges due to hydrogel‐based matrix visualization limitations, the preserved cellular footprints and extracellular matrix integrity provide compelling evidence of the authentic biomimetic arrangement.^[^ ^72^ ^]^ Furthermore, our findings underscore the 3D‐IVM‐HC model's robust capacity to replicate critical physiological processes, including epithelial differentiation, barrier formation, and crypt morphogenesis, without the need for external mechanical stimuli, highlighting the essential role of geometric cues in epithelial cell maturation and polarization.^[^ ^73^ ^]^

#### 2.1.4 Functional Analysis of Bioprinted Colon Tissue and Simulated Functional Assessment of 3D‐Bioprinted Colon Constructs versus Human Colon

**Figure 4:**
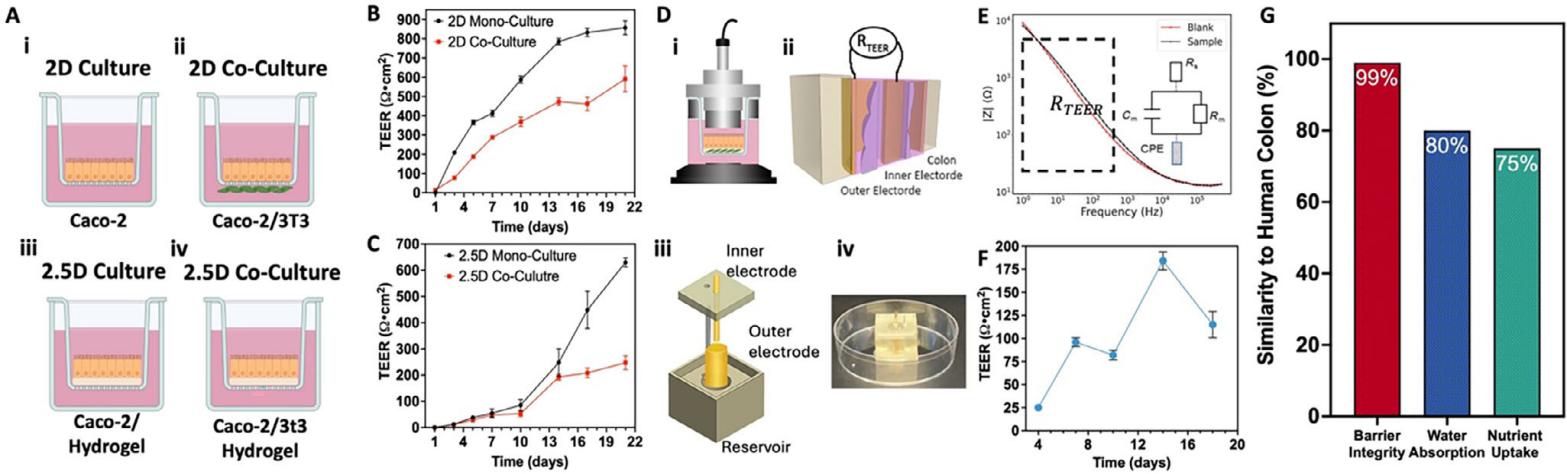
Validation of the 3D‐IVM‐HC model. A) Schematic representations of the various culture systems: 2D culture, 2D coculture with 3T3 fibroblasts, 2.5D culture on hydrogel disk, and 2.5D coculture with 3T3‐laden hydrogel disk setups. B) TEER measurements of Caco‐2 cells in monoculture and coculture with 3T3 fibroblasts in 2D Transwell systems over 21 days showed a higher resistance in monocultures. C) TEER measurements of 2.5D Caco‐2 cells in monoculture and coculture with 3T3 fibroblasts, indicating a reduction in TEER values for coculture systems. D) i, ii) Comparison of the TEER measurement setup for 2D/2.5D and our 3D‐IVM‐HC model. iii, iv) The design and assembled view of the measurement chamber with inner and outer electrodes within the reservoir. E) Impedance spectra (|*Z*|) of blank and sample colon constructs over a frequency range, highlighting the TEER region of interest. F) TEER values of Caco‐2 cells cultured in 3D‐IVM‐HC models from days 4 to 16, showing a peak in barrier function at around day 12. The results demonstrate the influence of 3T3 cells as stroma layer and 3D architecture matrices on Caco‐2 cell barrier integrity, with TEER values aligning more closely with in vivo conditions of the human small intestine. G) Comparison of the 3D‐IVM‐HC model with the native colon, based on in silico simulations evaluating three essential functions: barrier integrity, water absorption, and nutrient absorption. Each bar represents a single deterministic simulation result. Fluxes were computed using a 1D convection–diffusion formulation with permeability inferred from TEER via an equivalent‐circuit approximation; parameters were taken from the measured construct geometry and TEER/EIS.

To demonstrate our 3D‐IVM‐HC model's capability to create suitable 3D environments for cell culture, we assessed the barrier integrity of colon epithelium by measuring TEER over time.^[^ ^62^, ^71^ ^]^ Initial TEER evaluations were conducted on Caco‐2 cells, both in monoculture and cocultured with 3T3 fibroblasts, using Transwells in 2D and 2.5D formats (**Figure** **4**A). In the 2D setup, Caco‐2 cells were seeded on the apical side of the Transwell for monocultures, and for cocultures, fibroblasts were first seeded on the underside, followed by Caco‐2 cells on top. The 2.5D formats involved disk‐shaped hydrogels, both with and without embedded fibroblasts, serving as a base for Caco‐2 cell seeding in both mono‐ and cocultures. TEER measurements spanned from day 1 postseeding to day 21, utilizing blank Transwells and 3T3 cells alone as controls for monocultures and cocultures, respectively.

As depicted in Figure 4B,C, TEER values showed a consistent increase across the culture period for both hydrogel‐based and porous filter membrane setups, indicating the successful establishment of a functional epithelial barrier. Notably, the 2D Caco‐2 monocultures exhibited the highest TEER values, around 860 Ω cm^2^, which then decreased to below 260 Ω cm^2^ in 2.5D cocultures with 3T3. The integration of 3T3 cells and GelMA within the 2.5D cocultures was observed to modulate the characteristics of the Caco‐2 cell layer, resulting in reduced TEER values when compared to monocultures, thus emphasizing the subepithelial layer's role in tight junction modulation. This reduction in TEER values in coculture setups more accurately mirrors the in vivo small intestine TEER of 69 Ω cm^2^, suggesting a model that is more physiologically representative.

For the TEER measurements of the 3D‐IVM‐HC model, we employed a two‐point bioelectronics system, fitting the hollow structure between two electrodes to monitor impedance changes throughout the culture period (Figure 4D‐i,ii; Notes S9 and S10 and Figures S9 and S10, Supporting Information). A measurement chamber was specially 3D bioprinted to house the bioelectrodes, ensuring their consistent positioning during all measurements. The bioelectrodes' biocompatibility was verified, demonstrating minimal cytotoxic effects on cell growth and affirming their suitability for use (Figure S8, Supporting Information). From days 4 to 21 of culture, TEER measurements showed an increase in impedance in the colon construct with Caco‐2 cells, signifying a successful barrier formation (Figure 4E,F). Additionally, to further assess the integrity of the epithelial barrier, we analyzed electrical impedance spectroscopy (EIS) to evaluate the electrical properties of both blank samples and those including Caco‐2 cells, revealing significant insights into the barrier's development over time, with TEER values similar than the reported ex vivo^[^ ^74^ ^]^ (Figure 4D‐iii,iv). These comprehensive findings underscore our 3D‐IVM‐HC model's effectiveness in emulating the physiological barrier properties of the colon epithelium, highlighting its potential as a valuable tool in intestinal tissue engineering.

To establish our 3D‐IVM‐HC model as a practical platform for disease modeling and therapeutic assessment, we performed in silico simulations (Figure 4G; Note S10, Supporting Information) that focused on three pivotal colonic functions—barrier integrity, water absorption, and nutrient uptake. These processes collectively govern colonic homeostasis by controlling molecular passage, maintaining fluid and electrolyte equilibrium, and providing essential substrates for cellular metabolism. From a disease perspective, any disruption in barrier function, dysregulated fluid absorption, or aberrant nutrient flux can be a hallmark of gastrointestinal pathologies, including colorectal cancer.^[^ ^61^, ^75^ ^]^ Crucially, barrier integrity not only limits pathogen infiltration but also influences how chemotherapeutic agents diffuse into tumor sites, while water transport recreates the native luminal environment that impacts local drug distribution.^[^ ^2^, ^20^ ^]^ In addition, sustaining nutrient uptake is vital for modeling tumor growth dynamics, as cancer cells rely on adequate nutrient supply to proliferate.^[^ ^24^, ^76^ ^]^ By demonstrating that these core physiological attributes align well with in vivo benchmarks, we validate our 3D‐IVM‐HC model's relevance for accurately capturing tumor–drug interactions. Building on these foundational capacities, we next explored the construct's potential to foster tumor development and effectively evaluate chemotherapeutic regimens, thereby underscoring its utility as an integrative system for colorectal cancer research and treatment optimization.

Modeling note—in the current study, simulations are designed to capture the trends. Future work will experimentally validate flux predictions by quantifying apparent permeability of paracellular tracers and epithelial nutrient uptake, and benchmark these against established barrier‐model literature to refine model parameters. To estimate transport, we implemented a deterministic 1D convection–diffusion model parameterized by the measured lumen geometry and an equivalent‐circuit link between TEER and paracellular permeability. Such simplified frameworks are standard for epithelial barriers and intestinal absorption, enabling first‐order flux predictions without overfitting.^[^ ^77^, ^78^, ^79^ ^]^

#### 2.1.5 Generation of 3D Colon Cancerous Model and Study of the Effect of 5‐Fluorouracil (5FU) Treatment on the 2D‐ and 3D‐IVM‐HC Model

Replicating the native structural and functional complexity of colon tissue has remained a persistent challenge in colorectal cancer (CRC) research and drug development.^[^ ^3^, ^42^ ^]^ In this study, we establish a novel cancer model that mimics the in vivo tumor environment for effective drug screening, in this section, we reintroduce the use of HCT116 colorectal adenocarcinoma cell line.

**Figure 5:**
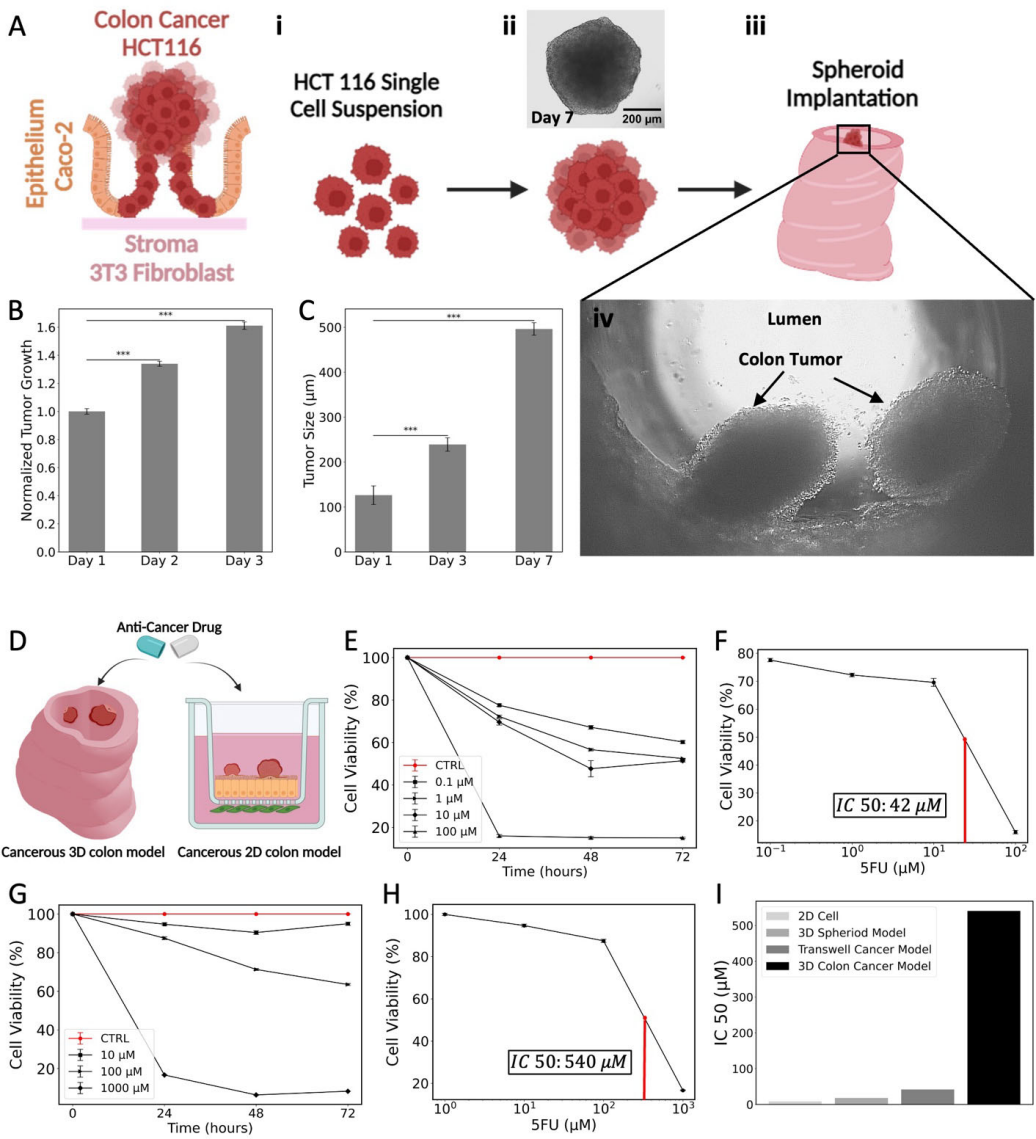
Development and validation of a 3D‐IVM‐HC cancer model for drug screening. A) Schematic of the epithelium–stroma interface with HCT116 colorectal adenocarcinoma cells. i–iii) Stepwise development of HCT116 spheroids from single‐cell suspension to spheroid implantation into the 3D‐IVM‐HC model. iv) Bright‐field image of the implanted tumors within the lumen. B) Diameter growth of HCT116 spheroids maintained in suspension culture for 7 days prior to implantation (bright‐field microscopy, mean ± standard deviation (SD), *n* = 20 spheroids). C) Bright‐field microscopy of HCT116 spheroids implanted into the lumen of the 3D‐IVM‐HC construct, monitored over three days prior to drug screening. D) Schematic showing the exposure of both Transwell and 3D in vivo mimicking colon cancerous models to 5FU. E) Dose‐dependent cell viability of the Transwell cancer model over time upon 5FU treatment at 0.1–1–10–100 µm. F) Determination of IC_50_ value for the Transwell cancer model by nonlinear regression. G) Cell viability of the 3D‐IVM‐HC cancerous colon model over time with varying concentrations of 5FU, at 10–100–1000 µm. H) IC_50_ value estimation for the 3D‐IVM‐HC cancerous model, indicating a significant increase compared to the Transwell model. I) Comparative IC_50_ values across different model formats, highlighting the enhanced chemoresistance in 3D‐IVM‐HC models. These results support the use of 3D‐IVM‐HC cancer models as more representative of in vivo conditions for cancer progression studies and drug efficacy screening. Compared to conventional flat culture systems and gut‐on‐chip devices, which predominantly model planar epithelial monolayers, the 3D‐IVM‐HC introduces a curved lumen‐like geometry that more closely parallels the native colon. This architecture supports spontaneous epithelial folding into crypt‐like domains, higher cell density, and enhanced polarization. In addition, the stromal–epithelial layering within the 3D matrix contributes to more realistic barrier function and drug response, as reflected by the clinically relevant chemoresistance observed against 5‐fluorouracil. These advantages underscore how macroscale luminal curvature and multilayer organization provide complementary insights beyond those attainable with flat culture platforms.^[^ ^77^, ^78^, ^79^ ^]^

To simulate the tumor microenvironment more accurately, we cultured HCT116 cells to compact form tumor spheroids, achieving a size that reflects the ratio between the colon lumen and actual colon cancer polyps.^[^ ^2^, ^16^ ^]^ Utilizing the hanging drop method, HCT116 cells aggregated into spheroids, ≈500 µm in diameter, over 7 days (**Figure** **5**A,B; Notes S12 and S13 and Figures S11 and S12, Supporting Information), aiming to represent the size of a stage I native tumor (5–10 mm).

As controls for our 3D‐IVM‐HC model, we employed several models including monolayer culture: HCT116 cells grown in standard 2D culture conditions for baseline comparison; tumor spheroids: formed using the hanging drop method to better mimic the 3D tumor environment; and Transwell model with cocultured fibroblasts: seeding fibroblasts on the underside of the Transwell, with Caco‐2 cells and then HCT116 spheroids on the epithelial layer, cultured for an additional three days to replicate the tumor–stroma interaction.

In our experimental setup, we first 3D bioprinted a 3T3‐laden hydrogel into a colon‐like architecture. After 7 days of culturing, Caco‐2 cells were seeded within the hollow part of this construct and allowed to attach and grow for another 14 days before implanting the HCT116 cancer spheroids. Bright‐field images show the colon construct with implanted cancer spheroids one day postimplantation (Figure 5A‐iv). Tumor growth rates within the colon construct were assessed by measuring the metabolic activity of samples due to the growth of cancer spheroids for three days postimplantation (Figure 5C).

To evaluate drug efficacy, we exposed both Transwell and 3D‐IVM‐HC colon cancer models to relevant intratumoral concentrations of 5FU.^[^ ^45^, ^80^ ^]^ Cell viability was quantified from 0 to 72 h posttreatment with escalating concentrations of 5FU, using a metabolic activity assay (Figure 5D). The Transwell model exhibited a significant dose‐dependent reduction in metabolic activity at 24 h exposure to 5FU (Figure 5E). The IC_50_ value, representing the drug concentration needed to inhibit a biological process by 50%, is a key parameter in pharmacology for evaluating drug potency. In this study, IC_50_ measurements were used across different models to examine the impact of the 3D microenvironment on the chemotherapeutic response. The Transwell model's IC_50_ was ≈42 µm, significantly higher than in monolayer cultures of HCT116 and HCT116 spheroids, pointing to the stromal layer's role in fostering chemoresistance (Figure 5F,I; Note S14 and Figure S13, Supporting Information).

The 3D‐IVM‐HC architecture model's response to 5FU demonstrated a dose‐dependent decrease in tumor cell metabolic activity, with an IC_50_ of ≈540 µm, indicating a substantial increase in drug resistance compared to the control models (Figure 5G,H). These findings emphasize the 3D colon architecture's superiority in mimicking in vivo conditions, offering a more accurate platform for studying cancer progression and screening drug efficacy. This analysis highlights the necessity of using 3D in vivo mimicking models that closely replicate the tumor microenvironment for enhanced comprehension and treatment of colorectal cancer.

The incorporation of HCT116 colon carcinoma spheroids into the construct reveals the model's impressive capability to authentically capture complex tumor–stroma interactions^[^ ^2^, ^58^ ^]^ and replicate drug‐response dynamics characteristic of clinical CRC, exemplified by enhanced chemoresistance to 5FU compared to traditional 2D cultures and simpler 3D models.^[^ ^29^, ^35^, ^41^ ^]^ Critically, our findings demonstrate that the 3D‐IVM‐HC model can significantly advance the understanding of cancer–stroma crosstalk in 3D, facilitating superior predictive evaluations of therapeutic responses compared to other existing models.^[^ ^40^, ^69^ ^]^ This comprehensive 3D integration of cellular architecture, mechanical properties, and biological interactions sets a new benchmark in disease modeling, significantly enhancing the accuracy and translational relevance of preclinical drug testing.^[^ ^2^, ^29^, ^39^ ^]^

Compared to any previously reported intestinal model, our 3D‐IVM‐HC model offers a uniquely scalable, customizable, and physiologically relevant framework, bridging longstanding gaps in tissue engineering. Its superior fidelity to human colon tissue positions it not only for advanced CRC studies but also for broader gastrointestinal applications, including inflammatory bowel disease, host–microbe interactions, and personalized medicine approaches. The future incorporation of additional cell types, immune components, and dynamic mechanical stimuli promises to further enhance its physiological relevance, expanding its applicability to even more complex gastrointestinal pathologies. Additionally, our 3D‐IVM‐HC model effectively addresses significant practical and ethical challenges associated with animal‐based research. Traditional animal models are well‐known for their extensive resource requirements, involving considerable financial expenditures, extended study durations, and intricate regulatory oversight. By contrast, our human‐cell‐based, animal‐free 3D‐IVM‐HC model significantly reduces these experimental costs—by around 70–80%—by eliminating expenses related to animal procurement, housing, and regulatory compliance.^[^ ^1^, ^2^, ^6^, ^16^ ^]^ Moreover, this approach shortens experimental timelines, enabling faster, more affordable, ethically responsible, and easily scalable translational studies. Crucially, by removing interspecies variability, the 3D‐IVM‐HC model is anticipated to substantially improve clinical relevance and translation, offering an expedited and ethically sound route for preclinical investigations. Collectively, our 3D‐IVM‐HC model demonstrates the transformative potential in faithfully recreating essential structural and functional aspects of the 3D human colon. By bridging the critical gap between simplified in vitro models and the intricate dynamics and heterogeneously of human physiology, our 3D‐IVM‐HC model can potentially significantly advance the field, enabling more accurate mechanistic studies, better predictive drug assays, and novel regenerative medicine strategies for gastrointestinal diseases.

## 3 Conclusion

In conclusion, our novel biomimetic 3D‐IVM‐HC model represents a transformative advancement in disease modeling and precision drug discovery. By accurately replicating the structural, mechanical, physiological, chemical, and biological complexities inherent in human colon tissue, this innovative inv‐ivo mimicking 3D model addresses the significant limitations of conventional in vitro and in vivo models. Through spontaneous formation of crypt‐like domains and precise replication of luminal curvature, mucosal architecture, and layered tissue organization, our 3D‐IVM‐HC model closely mirrors the heterogeneous microenvironment of the native human colon. Comprehensive characterization and validation underscore its superior physiological relevance, demonstrating enhanced capabilities in modeling colorectal cancer progression and accurately evaluating therapeutic responses. Ultimately, this first‐of‐its‐kind 3D‐IVM‐HC non‐animal model significantly enriches our understanding of colorectal cancer biology and propels the advancement of personalized medicine, paving the way for highly effective precision therapeutics.

## 4 Experimental Section

### Materials

In this study, gelatin type B, calcium chloride dihydrate, agarose, and ammonium hydroxide were purchased from Fisher Chemical. Pluronic F‐127, gum Arabic, alginic acid sodium salt, Triton X‐100, and 5‐fluorouracil were purchased from Sigma‐Aldrich. Dulbecco's Modified Eagle's Medium (DMEM), fetal bovine serum (FBS), penicillin–streptomycin, and trypsin were obtained from Gibco. Live/Dead viability assays were obtained from Invitrogen (CA, USA). Bovine serum albumin was obtained from Fisher Bioreagents.

### Preparation of Gelatin Support Bath

FRESH support bath was prepared following the procedure reported earlier by Lee et al. Ref. [51] In brief, first, 2.0% w/v gelatin Type B (Fisher Chemical), 0.25% w/v Pluronic F‐127 (Sigma‐Aldrich), and 0.1% w/v Gum Arabic (Sigma‐Aldrich) were dissolved in a 50% v/v ethanol solution at 45 °C in a 1 L beaker. To form the slurry of gelatin microparticles, the beaker was then placed under an overhead stirrer, sealed with aluminum sheet to minimize evaporation, and allowed to cool to room temperature while stirring for 8 h. The resulting slurry was then divided into 50 mL conical tubes and centrifuged at 300 *g* for 5 min to compact the gelatin microparticles. The supernatant was then removed, and gelatin microparticles were resuspended in a washing solution (0.2% w/v CaCl_2_ (Fisher Chemical)) to remove the ethanol and Pluronic F‐127. The gelatin slurry was then washed 3 times with a washing solution at 1000 *g* for 2 min. Prior to printing, the uncompacted slurry was degassed in a vacuum chamber for 15 min, followed by centrifugation at 2000 *g* for 10 min with the temperature of centrifuge set to 10 °C. The supernatant was removed, and the gelatin slurry was transferred into a print container of appropriate volume, typically 50% larger than the construct to be bioprinted. For sterile support preparation, all hardware was either cleaned with 70% ethanol and UV sterile inside the biosafety cabinet.

### Bioink Preparation

Bioinks were prepared by dissolving in a sterile environment GelMA (Advanced Biomatrix, USA) in phosphate‐buffered saline (PBS) in a concentration of 7.5% w/v, 0.2% w/v of LAP (Allevi, USA) as the photoinitiator and 0.5% alginate w/v and homogenizing it by magnetic stirring at 300 rpm overnight.

To enable visualization of printed constructs by confocal and light‐sheet microscopy, fluorescent microparticles were incorporated into the bioinks. The preparation and washing of the particles followed previously published protocols.^[^ ^2^, ^56^, ^81^, ^82^ ^]^ Briefly, fluorescent particles were purified by repeated centrifugation and resuspension in deionized water before incorporation into GelMA/alginate inks at a ratio of 10 µL suspension per 1000 µL bioink. Because the particles remained solid and did not dissolve, they had minimal effect on viscosity or printability.

### Characterization of Bioink—Rheology

The rheology profile of the GelMA–alginate bioink was characterized by using a TA DHR‐2 Discovery series (TA instruments, UK) equipped with a Peltier for temperature control. Parallel plate geometry (25 mm) was used with a gap of 0.3 mm. GelMA–alginate ink was previously tempered at 37 °C before each test. The shear rate effect in the viscosity of GelMA was measured at a constant temperature of 25 °C by varying the shear rate between 0.01 and 1000 s^−1^ in the rotational mode. The sweep tests of the GelMA–alginate ink were evaluated with frequencies ranging from 0.1 to 10 rad s^−1^ at a rotation amplitude of 1% at 25 °C.

### Characterization of Bioink—Compressive Modulus

To study the compression modulus of the GelMA–alginate bioink, samples were prepared and molded with a diameter of 15.6 mm and a height of 5 mm. Samples were cross‐linked using the same dual‐cross‐linking protocol as applied to the 3D‐printed constructs: ionic cross‐linking in 0.2% CaCl_2_ for 20 min followed by UV photo‐cross‐linking (405 nm, 1 min). This approach was selected to reproduce the cross‐linking kinetics and conditions of the printed colon constructs as closely as possible. The unconfined compression test was performed using an Instron 3365 UTS testing machine (Norwood, MA, USA) at room temperature under a compression rate of 200 µm min^−1^. The mechanical properties of compression were obtained from the averaged compression stress–strain curve. The compression modulus was calculated as the slope of the linear region in the 0.1–0.15 strain range of the stress–strain curves.

### Cell Culture

HCT116 and Caco‐2 cells were obtained from ATCC, and 3T3 fibroblasts were generously donated by Prof. Benavente from the Department of Pharmaceutical Sciences at the University of California, Irvine, CA. Briefly, HCT116 and 3T3 cells were cultured in DMEM and the Caco‐2 cells were cultured in Eagle's Minimum Essential Medium (EMEM). The culture media for all cell lines were supplemented with 10% v/v FBS and 1% v/v penicillin–streptomycin. Cells were incubated at 37 °C in a 5% CO_2_ atmosphere. All cells were used in passages lower than 20.

### Cell Culture on Transwell Inserts

First, Transwell inserts (Corning, USA) were incubated in culture media for 2 h to equilibrate the membrane for seeding. For 2D monocultures, Caco‐2 epithelial cells were seeded onto the apical side of 24 mm Transwell inserts, as shown in Figure 5, at 2.5 × 10^5^ cells per well density. The cells are cultured in 1 mL of culture medium in the basolateral compartment and 400 µL into the apical compartment. For coculture experiments, 3T3 fibroblasts were initially seeded followed by Caco‐2 addition. Transwell inserts were initially inverted and seeded with 1 × 10^5^ cells in 50 µL of 3T3 medium and incubated for 1 h to encourage cell attachment to the membrane. Thereafter, seeded inserts were returned to wells containing 1 mL of 3T3 medium and grown for 5 days. Four days after fibroblast seeding, inserts were seeded with Caco‐2 cells on the apical side as previously described. For 2.5D cultures in Transwell format, first, 100 µL of hydrogel with and without embedded 3T3 fibroblast at 5 million mL^−1^ was drop casted in 96 well plate and cross‐linked for 1 min at 405 nm UV exposure, later on, 200 µL of 0.2% CaCl_2_ was added to the well and let stay for 10 min. After cross‐linking, the hydrogels were transferred to the apical side of the Transwell and cultured for 5 days. After day 5, Caco‐2 cells at 2.5 × 10^5^ cells per well density were seeded on top of the hydrogels with and without 3T3 fibroblasts for mono‐ and coculture samples. Samples on Transwell inserts were fed every 2–3 days with 1 mL of culture medium in the basolateral compartment and 400 µL into the apical compartment. For some experiments, Transwell inserts seeded only with 3T3 fibroblast on the basolateral side were used and referred to as fibroblast monoculture on Transwell inserts.

### 3D Bioprinting Protocol and Construct Culture

In preparation for printing the double‐layer colon model, Colon NIH 3D model was processed to generate a scaled‐down computer‐aided design (CAD) model (10 mm in length and 5 mm in diameter) composed of two interconnected layers that mimicked the cancer tumor and the external epithelium. The STL design was generated from CT‐derived colon data and downscaled to dimensions of ≈10 mm in length and 5 mm in diameter. The construct was sliced into 100 layers in the *Z*‐direction for printing, yielding an average wall thickness of ≈400 µm. The inner hollow lumen was designed to accommodate epithelial seeding (Caco‐2 cells), while the outer layer was intended to encapsulate fibroblasts within the GelMA/alginate matrix. These parameters collectively preserved luminal curvature, wall‐to‐lumen ratios, and overall architecture consistent with the native colon, ensuring structural fidelity during culture. Later, the CAD model was sliced and processed using Cellink HeartWare software (CellInk, USA) to generate the G‐code. Bioink formulations were optimized to meet the mechanical properties and cell viability of colon tissues. HCT116 and 3T3 cells were dispersed, respectively, at concentrations of 3 × 10^6^ and 8 × 10^6^ cells mL^−1^, in the early described bioinks. Then, cell‐laded bioinks were transferred to extrusion cartridges avoiding the formation of bubbles, and G22 nozzles were attached. To print the colon model, embedding extrusion bioprinting was performed (FRESH method), by using an Inkredible+ bioprinter (CellInk, USA). In preparation, the sterilization of the printer was performed by using ethanol 70%, UV light, and using the HEPA filter embedded within the bioprinter. Once sterilized, the cartridges were placed inside the printhead along with the reservoir filled with a gelatin support bath. The cartridges and needles were positioned in the *XY* center of the container. The bioprinting was then started. All colon constructs were bioprinted at 27 °C. Upon bioprint completion, colon constructs were exposed to 405 nm light for 1 min to cross‐link the GelMA. Later, the container was incubated at 37 °C to melt the gelatin support bath and release the bioprinted colon. Once released, colon constructs were washed with fresh media to remove the melted gelatin support bath and then cultured.

### Construct Staining and Embedding—Life/Death Assay

The LIVE/DEAD assays were conducted following the manufacturer's instructions. Briefly, the culture medium was removed from bioprinted constructs, and two PBS washes were performed to extract the remains of the media. The LIVE/DEAD reagent was then added to the samples and incubated for 40 min in the incubator, followed by three washes with PBS. Finally, the samples were examined by LSM 900 with Airyscan Microscope (Carl Zeiss, Jena, Germany).

### Construct Staining and Embedding—Immunofluorescence Staining

Immunostaining was performed by fixing the colon bioprinted samples with 4‐paraformaldehyde (Fisher Scientific, Waltham, MA, USA) for 60 and 10 min for Transwell format samples, followed by two washes with 1× PBS. After washing, cell permeabilization was done using 0.1% v/v Triton X‐100 in PBS for 20 min and blocking was performed with 3% BSA in PBS to prevent nonspecific binding. F‐actin cytoskeleton was stained by incubating bioprinted constructs with Alexa Fluor 488 Phalloidin (Thermo Fisher Scientific, USA) (1:40 dilution), tight junction proteins were stained by Alexa Fluor 555 conjugated ZO‐1 (Invitrogen, USA) (5 µg mL^−1^ in blocking buffer) at 4 °C overnight. The optimal concentration used for antibodies were based on the manufacturer's instructions. Samples were washed with PBS 3 times and then stained with DAPI for 20 min at room temperature. After washes with PBS, the samples were embedded in agarose gel and sectioned as explained below and were imagined by LSM 900 with Airyscan Microscope (Carl Zeiss, Jena, Germany) and Z.1 light‐sheet microscopy (Carl Zeiss, Jena, Germany).

### Construct Staining and Embedding—Agarose Embedding

For fluorescence microscopy imaging, the bioprinted colons required to be sliced in small cross‐sections. To provide mechanical support during the cutting, the stained colon bioprinted constructs were embedded in agarose (Figure S7, Supporting Information). Briefly, agarose was dissolved in distilled water to produce a 1% w/v solution at 60 °C under magnetic stirring for 2 h. Once dissolved, agarose was cooled down to 40 °C for embedding. The barrel and plunger of a 3 mL syringe were disarmed. The stained colon construct was gently placed inside the barrel with the help of a spatula, and the plunger was carefully reinserted to not damage the sample. Agarose was then absorbed slowly until covering the full construct, then the syringe was placed vertically until the agarose solidified. The top of the syringe was cut to release the stained embedded colon sample. Finally, a pathology blade was then used to slice the embedded construct.

### Electrode and Chamber Fabrication

To ensure uniform spacing between electrodes, a specialized chamber was designed to house both the reference electrode and the working electrode. Utilizing Solidworks (Solidworks Corp., USA), the chamber was conceptualized as a cylindrical structure with an inner diameter (DI) of 6 mm and a height of 10 mm (Figure S9, Supporting Information). The upper section of the chamber served as a lid, incorporating a central aperture designed to secure the second electrode, which had a DI of 4 mm. The design facilitated a key‐and‐lock mechanism between the lid and the chamber body, ensuring the electrodes' positions remained stable throughout the measurement process. Details of the chamber's design, including specific dimensions, are provided in Figure S10 (Supporting Information). The fabrication process of the chamber involved 3D printing using a LumenX digital light processing printer (Cellink, USA) with a plastic resin. The design was prepared for printing by slicing it into layers with a height of 100 µm. Postprinting, both the chamber and lid underwent a thorough cleaning to remove any residual resin, followed by ultraviolet curing to ensure complete solidification. Subsequently, the chamber was affixed to a petri dish using adhesive and allowed to dry overnight. For the electrodes, a PI–Gold flexible sheet (KOYE Technology, China) was used, which was precisely cut using a cutter plotter and folded to fit within the chamber and through the lid's aperture. To enhance adhesion between the electrodes and their respective surfaces, double‐sided tape was employed. This methodological approach ensured a consistent and reliable setup for electrochemical measurements, highlighting the precision and care taken in the fabrication of the experimental apparatus.

### Measurement of TEER

The TEER of both Caco‐2 cell monoculture and Caco‐2/3T3 coculture cell models housed in 24‐well Transwell inserts was measured using an EVOM (World Precision Instruments, USA). A blank Transwell insert with no cells and only hydrogel served as a blank control for the monoculture model while a Transwell insert with 3T3 cells with and without hydrogel layer served as a blank control for the coculture model. TEER values (Ω cm^2^) of cell models were calculated using Equation (1)

(1) $$TEER=\left(R-R_{blank}\right)A$$

where *R* is the resistance measured across the cell layer(s), *R*_blank_ is the resistance of a blank as explained earlier, and *A* is the surface area of a Transwell insert (0.33 cm^2^).

For the 3D colon construct measurements, first the fabricated electrode and chamber were sterilized using 70% ethanol and UV sterilized for 30 min. Then, the electrodes were connected to potentiostat (VersaSTAT 3, Princeton Applied Research) in two‐electrode measurement setup, the applied DC voltage between the two electrodes was set to 0 and 0.01 V, AC voltage was applied for the measurements. After that, 1× PBS was used to check the stability of the setup and data were recorded from 1 to 10^6^ Hz. After that, the samples without Caco‐2 cells were measured as the blank measurements and later on the colon samples with Caco‐2 cells were measured through culture time from days 1 to 17. The impedance differences associated with the growth of Caco‐2 cells were calculated based on the averaged difference between black and sample at 10 to 1 kHz. The values were multiplied by the surface area of the lumen part of the colon (1.25 cm^2^) to find the resistivity of the Caco‐2 layer.

### Hanging‐Drop Method for Spheroid Formation

HCT116 spheroids were generated using the hanging‐drop technique. Briefly, a concentration of 5 × 10^4^ cells per drop was used. Drops of 52 µL were deposited in a petri dish and carefully flipped to produce a hanging drop meniscus, as depicted by Figure S9 (Supporting Information). To avoid evaporation, 4 mL of PBS was added into the lid, and the petri dish was sealed with parafilm. The spheroids were cultured in an incubator, and their growth was monitored daily with an inverted microscope through culture (Figure S11, Supporting Information). The size distribution of the spheroid was analyzed from optical microscopy images.

Mature HCT116 spheroids (≈500 µm in diameter after 7 days of hanging‐drop culture) were carefully transferred using a wide‐bore pipette tip into the lumen of preseeded 3D‐IVM‐HC constructs. Each construct received two spheroids placed in direct contact with the differentiated Caco‐2 epithelial layer to mimic tumor formation in the colonic lumen. Bright‐field microscopy was used to verify spheroid positioning and to monitor their integration into the construct over three days (Figure S12, Supporting Information). After this stabilization period, constructs were used for drug screening experiments with 5‐fluorouracil as described below.

### Metabolic Activity Analyses

The metabolic activity of the cells contained in the colon constructs were evaluated at days 0, 1, 2, and 3 of drug exposure. The samples were incubated with 10% v/v of Resazurin reagent (R&D Systems, USA) in EMEM media for 4 h at 37 °C. Then, the supernatant was collected and replaced for control samples with fresh culture media and for treated samples with media + drug. The fluorescence of the supernatant was measured at an excitation wavelength of 530 nm and an emission wavelength of 590 nm with a microplate reader (BioTek Cytation 5, Agilent).

### Statistical Analysis

The statistical analysis was conducted using analysis of variance in Python script. *p*‐Values (*p < 0.05, **p < 0.005, and ***p < 0.0005) were considered to be statistically significant. The data were presented with the standard deviation of at least three replicate experiments.

## Conflict of Interest

The authors declare no conflict of interest.

## Author Contributions

S.N. and J.A.T.‐N. contributed equally to this work. R.E. designed and conceptulized the study. S.N and J.A.T.‐N. developed the experimental setup and conducted the experiments. J.A.T.‐N., S.N., A.G., S.Z., and R.E. analyzed the data. S.N. and J.A.T.‐N. prepared the figures. J.A.T.‐N., S.N., S.Z. and R.E. wrote the first draft of the paper. A.G. developed some of the image analysis protocols. M.K., S.N. and A.A.performed the simulations. Q.S. provided some reagents and resources. M.K. provided help with fluorescent microscopy. R.E. supervised the study. All authors reviewed and edited the paper before submission.

## Supporting information



Supporting Information

Supplemental Movie 1

Supplemental Data

## Data Availability

The data that support the findings of this study are available from the corresponding author upon reasonable request.
