# Supplementary material for: Development of a 3D Human Colon Model Along with Bioelectronics for the Induction and Monitoring of Diseases
Source: Adv Sci (Weinh). 2025 Sep 27;12(45):e06377. doi: 10.1002/advs.202506377 (PMC12677662; doi:10.1002/advs.202506377)
Supplement: Supplementary file 1 — Supporting Information [file ADVS-12-e06377-s001.docx]

# Development of a 3D Human Colon Model Along with Bioelectronics for the Induction and Monitoring of Diseases

# Supporting Information Note 1 | Rheological behavior of four ink candidates

The rheological properties of bioink formulations of GelMA at 5% and 7.5%, and GelMA 7.5% mixed with 0.25% and 0.5% Alginate were meticulously evaluated. Fig. S1A illustrates the inherent shear thinning behavior of all bioink candidates, characterized by a viscosity decrement in response to escalating shear rates. The characteristics of this non-Newtonian fluid are crucial for extrusion-based three-dimensional printing, as they facilitate optimal printability through fine nozzles and ensure the structural integrity of the resultant constructs through prompt solidification^1^. Initial viscosity measurements at a shear rate of approximately 10 s−1 and a temperature of roughly 22°C yielded viscosities of 0.27, 0.30, 0.41, and 0.95 Pa*s for GelMA 5%, GelMA 7.5%, GelMA 7.5% with 0.25% Alginate, and GelMA 7.5% with 0.5% Alginate, respectively. These viscosities correlate directly with the increment in GelMA concentration and the integration of Alginate, serving as a rheological modifier. Further, the gelation kinetics of these inks were scrutinized by methodically diminishing the temperature at a uniform rate of 1°C per minute. The modified GelMA formulations, specifically those with alginate, manifested a pronounced increase in viscosity near 20°C, diverging from the unmodified GelMA 5%, which exhibited a comparable viscosity augmentation at approximately 16°C. These findings accentuate the distinctive gelation thresholds and viscosity responses of the bioinks in question (Fig. S1B). Moreover, our analysis included an assessment of the storage modulus (G') and loss modulus (G") over a range of oscillatory frequencies. The results indicated a dependency of both moduli on the frequency of oscillation, with alginate presence enhancing these rheological parameters. Consequently, this implies that alginate incorporation enables the fine-tuning of GelMA hydrogels' mechanical and viscoelastic characteristics, amplifying their applicability in bespoke bioprinting endeavors where precision-engineered material properties are imperative (Fig. S1C).


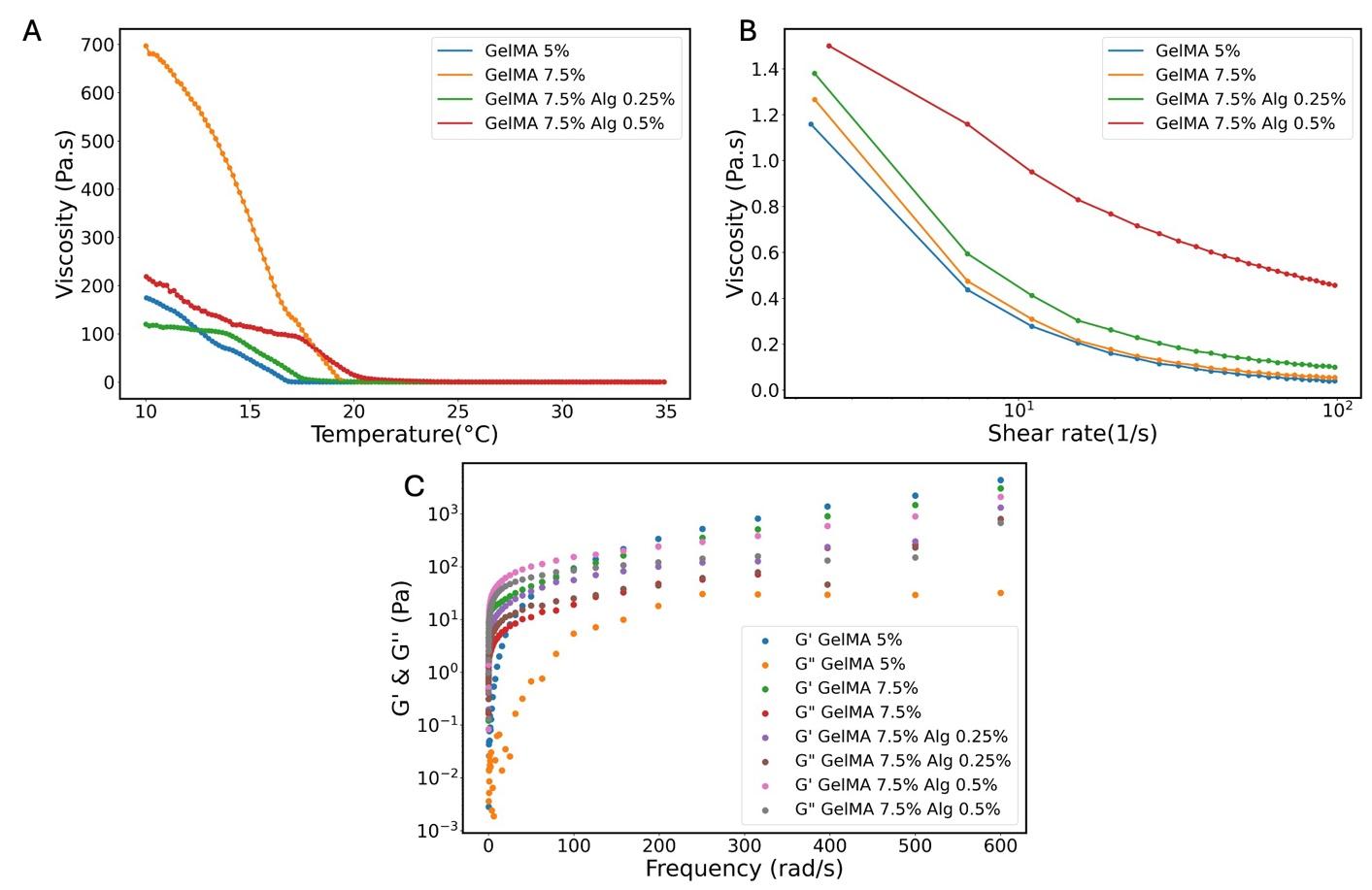


Fig S1. Rheological behavior of the GelMA at 5% and 7.5%, and GelMA 7.5% mixed with 0.25% and 0.5% candidate inks. A) Viscosity of bioinks as a function of temperature B) Viscosity of the candidate inks as a function of the shear rate. C) Storage (*G*′) and loss (*G*″) moduli of the candidate inks as a function of frequency.

# Supporting Information Note 2 | Fibroblast elongation in GelMA at 5% and 7.5%, and GelMA 7.5% mixed with 0.25% and 0.5% candidate inks

Hydrogel bioinks facilitate the investigation of cell-matrix interactions in settings that mimic physiological conditions. These developments demonstrate that mechanical stimuli, such as substrate stiffness, have a direct impact on fibroblast activation. Therefore, we observe the cell proliferation and elongation in the different bioink candidates using brightfield micrographs and the proliferation through the metabolic activity quantification by resazurin reduction. Phase contrast microscopy reveals the influence of hydrogel composition on cell morphology, aggregation, and network formation. Higher GelMA concentration coupled with higher Alginate concentration favors less cell clustering and promotes the formation of elongated fibroblast networks (Fig. S2 A-D). The metabolic activity illustrated in Figure S2E substantiates this observation, demonstrating that the metabolic activity was elevated in the bioink composed of 7.5% GelMA and 0.5% Alg over a span of seven days, as compared to its counterparts.


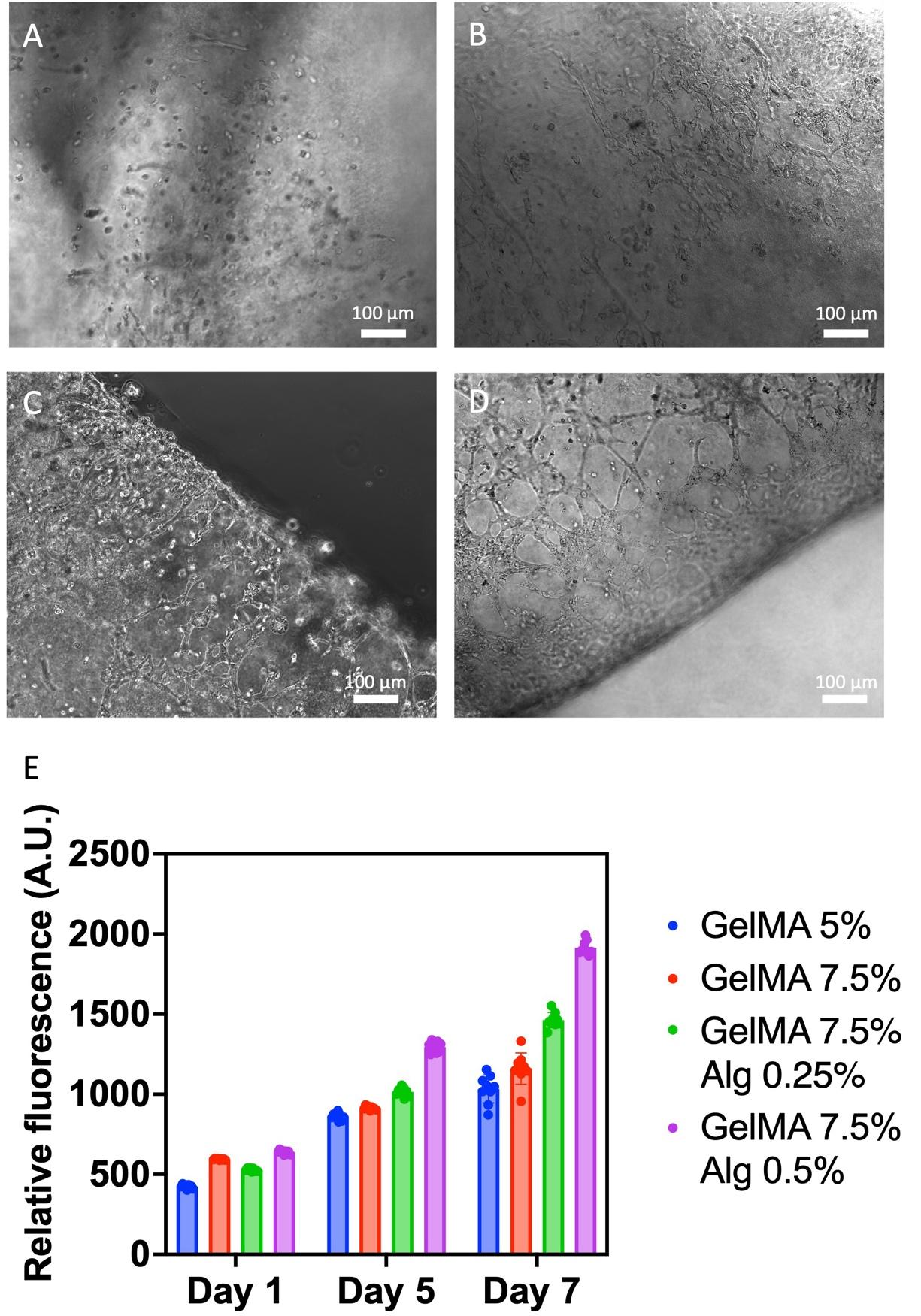


Fig. S2. Characterization of the elongation and proliferation of 3T3 fibroblasts embedded in the four bioink candidates. Optical microscopy images on day 7 of A) GelMA at 5%, B) GelMA 7.5%, C) GelMA 7.5% Alg 0.25%, and D) GelMA 7.5% Alg 0.5% bioinks. Scale bar: 100 μm. E) Metabolic activity of 3T3 fibroblasts cultured in the four bioink candidates, as measured by the Resazurin assay.

**Supporting Information Note 3 | Live/Dead assay in 3D bioprinted colons**

Figure S3 presents cross-sections of the printed colons stained with Calcein AM/Propidium Iodide (Live/Dead) to demonstrate cell survival on Days 1, 7, and 14. The sustained cell viability throughout the 14 days of culture suggests that the bioprinting process and the culture did not compromise the key cellular functions. Additionally, the printed colon's architectural integrity was maintained for up to 14 days.


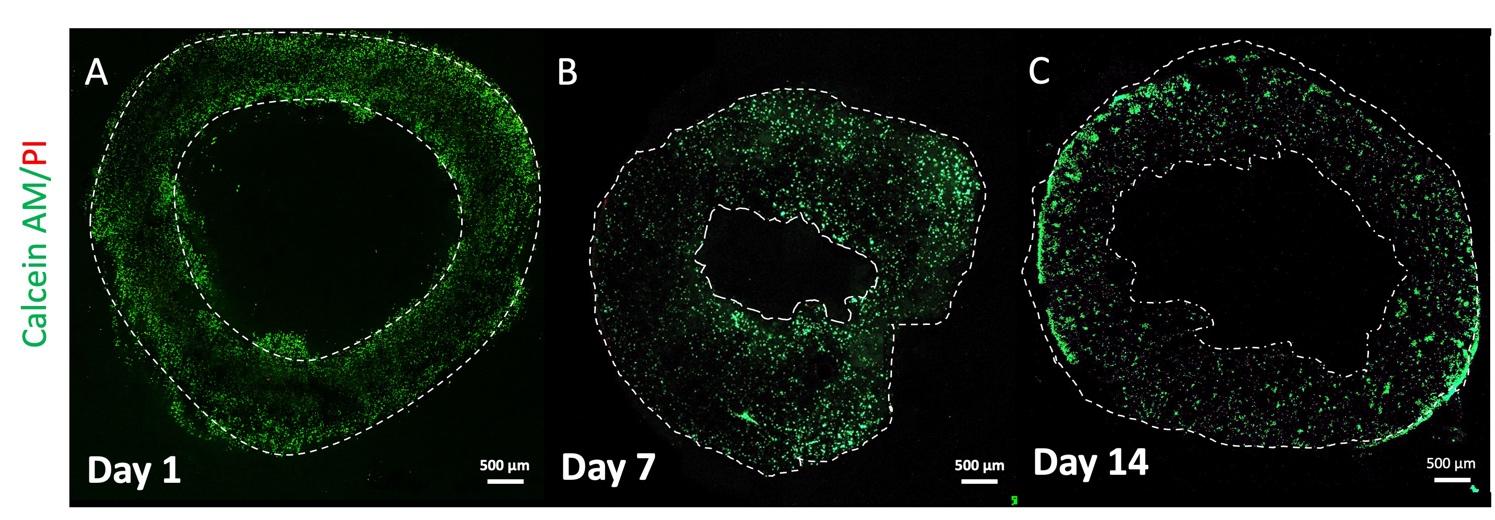


Fig. S3. Assessment of the cell viability in the bioprinted colon models with 3T3 fibroblast embedded in GelMa 7.5% Alg 0.5% at different time points; A) Day 1, B) Day 7, and C) Day 14.

**Supporting Information Note 4-6 | Characterization of HCT116 colon carcinoma spheroid formation inside the bioink**

The introduction of HCT 116 colon carcinoma cells to the internal layer of the double-layered construct allowed for the evaluation of the matrix's ability to support the growth of HCT 116 carcinoma cells. As shown in Fig. S4, Brightfield microscopy captured single and double cells on day 1. By day 7, spheroid formation was observed, with diameters ranging from 20 to 60 μm. By day 14, some spheroids reached diameters of up to 100 μm, maintaining this size until the conclusion of the experiment on day 21.


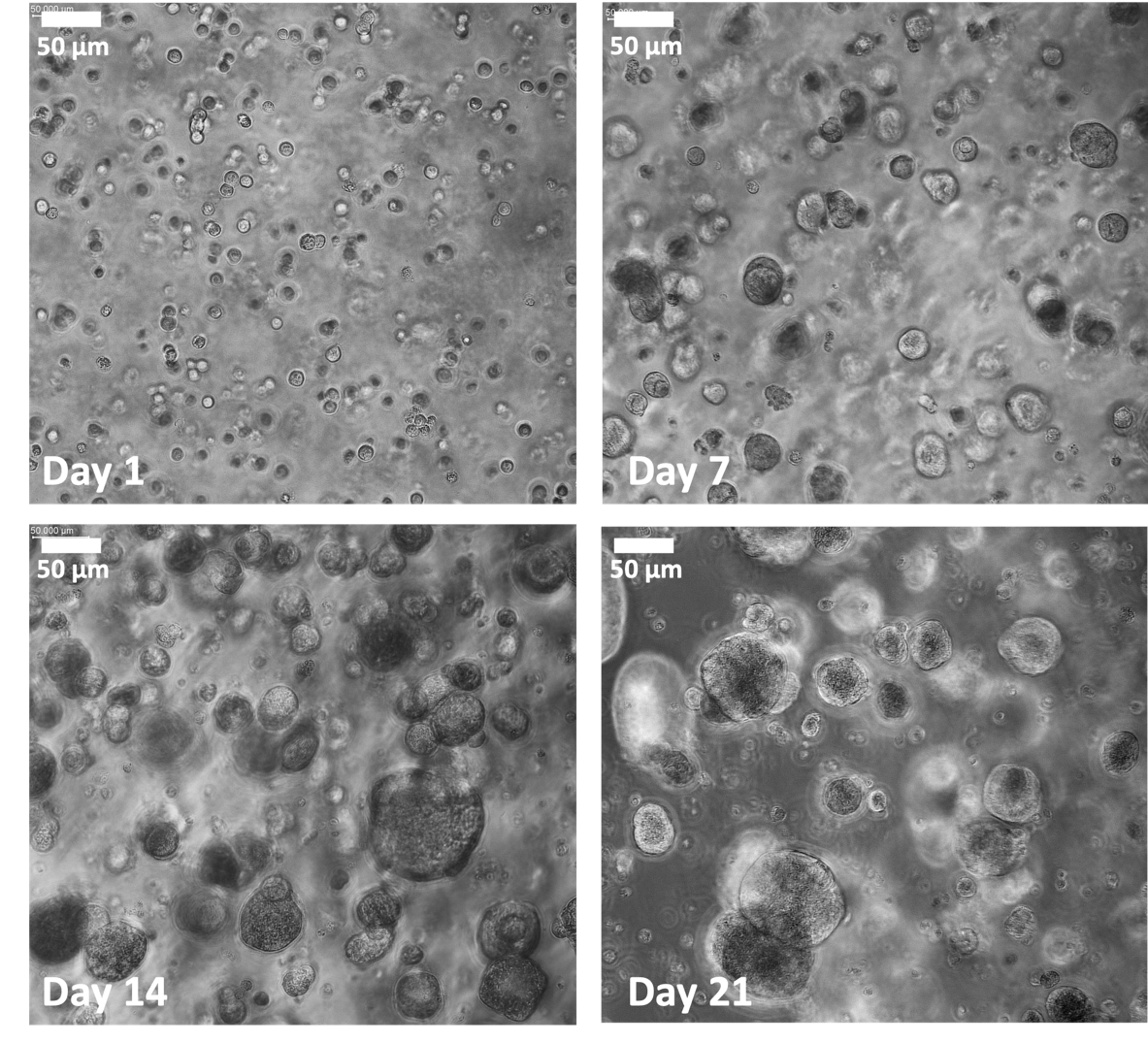


Fig. S4. Brightfield micrographs of HCT 116 cells embedded in the bioink, evaluated at days 1, 7, 14, and 21. Scale bar: 50 μm.

The viability of the HCT 116 spheroids was assessed using Calcein AM/Propidium Iodide staining on days 1, 14, and 21, as shown in Fig. S5.


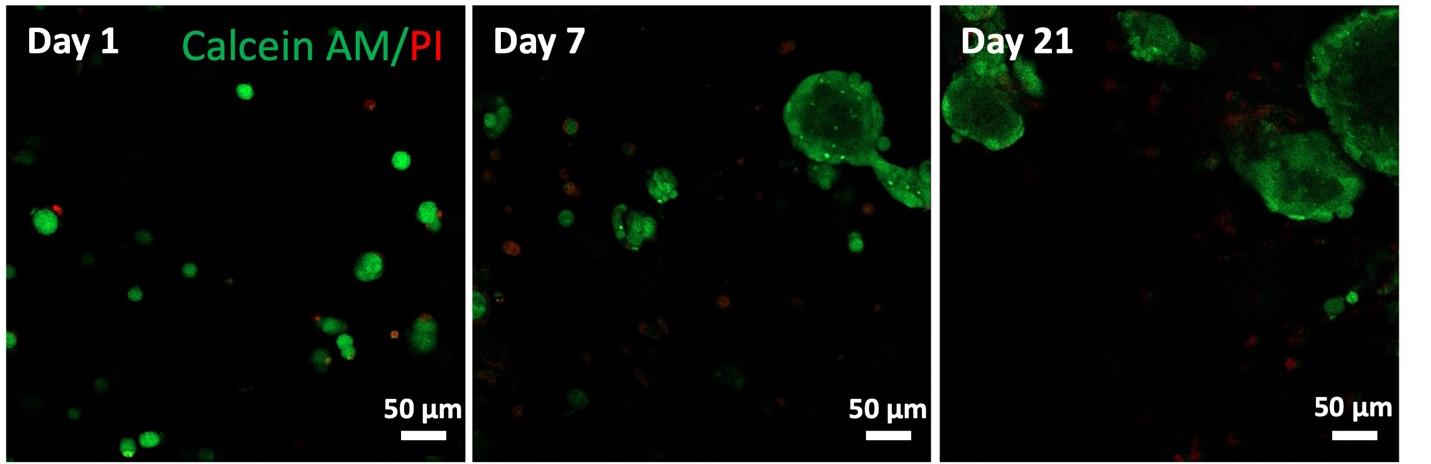


Fig. S5. Fluorescent micrographs of HCT 116 cells stained with Calcein-AM (green) to indicate live cells and Propidium Iodide (red) to indicate dead cells. Scale bar: 50 μm.

Additionally, the junctional integrity of the spheroids was evaluated by staining the tight junction protein ZO-1 on day 21. Confocal micrographs presented in Fig. S6 revealed organized tight junctions both on the surface and within the interior of the spheroids. DAPI staining confirmed a dense fluorescence of nuclei within the spheroids, indicating active cell proliferation and organization.


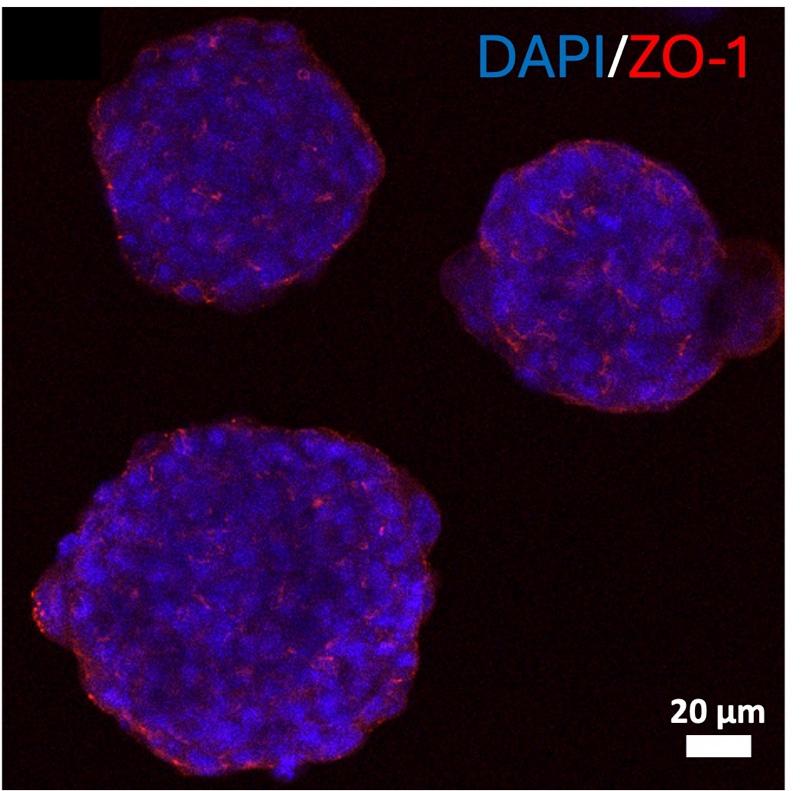


Fig. S6. Confocal micrograph of HCT 116 spheroids embedded in the bioink on day 21 stained with DAPI in blue and ZO-1 in red. Scale bar: 20 μm.

**Supporting Information Note 7 | Agarose Embedding of 3D Bioprinted Colons for Structural Support and Microscopy Sectioning**

The 3D bioprinted colons are constructed with walls approximately 400 μm thick and a height of 10 mm, posing challenges for confocal microscopy. To address this, a protocol was developed for embedding the colon samples in agarose, as shown in Fig. S7A. i-ii) The colon sample is first transferred into a 3 mL syringe. iii) A 1% agarose solution is drawn in to cover the structure, allowing it to gel fully. iv) Once the agarose solidifies, the plunger is pushed downward to separate the embedded structure from the syringe tip. v) The tip of the syringe is then cut off. v-vi) Finally, the embedded structure is released. The agarose-embedded colon sample is subsequently sectioned using a scalpel (Fig. S7B), and the sections are photo-documented under a microscope. The full embedded structure was also used for light-sheet microscopy to reconstruct the 3D distribution of cells in the printed colons.


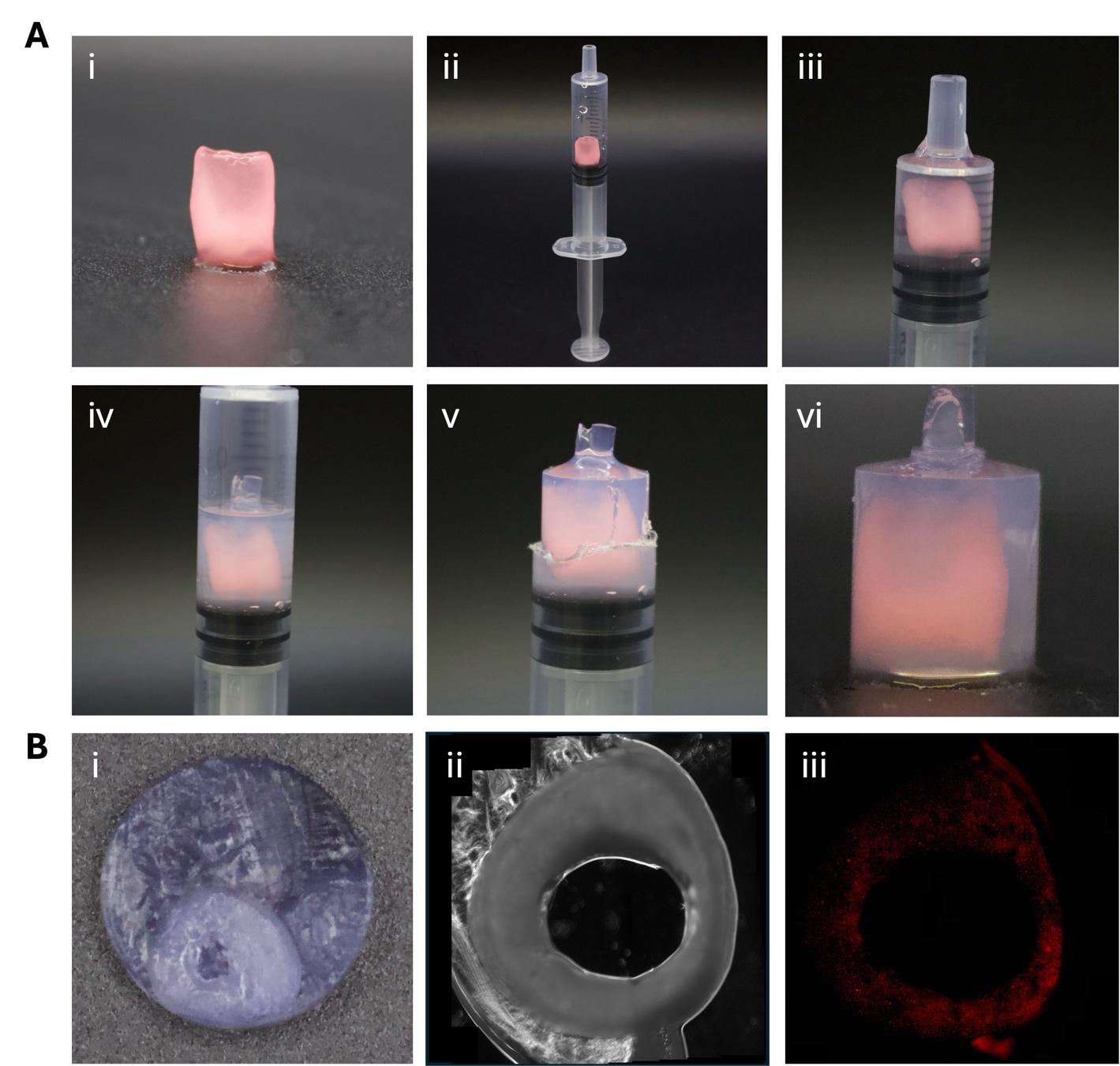


Fig. S7. Agarose embedding strategy for 3D bioprinted colons to facilitate microscopy. A) Embedding process. i-ii) The colon sample is transferred into a 3 mL syringe. iii) A 1% agarose solution is drawn in to cover the structure, allowing it to fully gel. iv) Once the agarose solidifies, the plunger is pushed downward to separate the embedded structure from the syringe tip. v) The syringe tip is cut off. v-vi) The embedded structure is released from the syringe. B) The embedded colon sample is sectioned using a scalpel, and the sections are photo-documented under a microscope.

# Supporting Information Note 8 | Immunostaining of the Caco-2 cells seeded on the inner wall of the 3D bioprinted colon model

# To verify the functionality of the epithelial layer formed from the Caco-2 cells in the inner wall of the 3D bioprinted colon model, we assessed the immunofluorescence analysis of intestinal epithelial differentiation in the model (Fig S.8). The expression of tight junction proteins, particularly Zonula occludens-1 (ZO-1), confirms the successful differentiation of the intestinal epithelium. The immunofluorescent staining, which shows cell nuclei in blue, F-actin in green, and ZO-1 in red, highlights tight junction formation within the scaffold lumen.

#
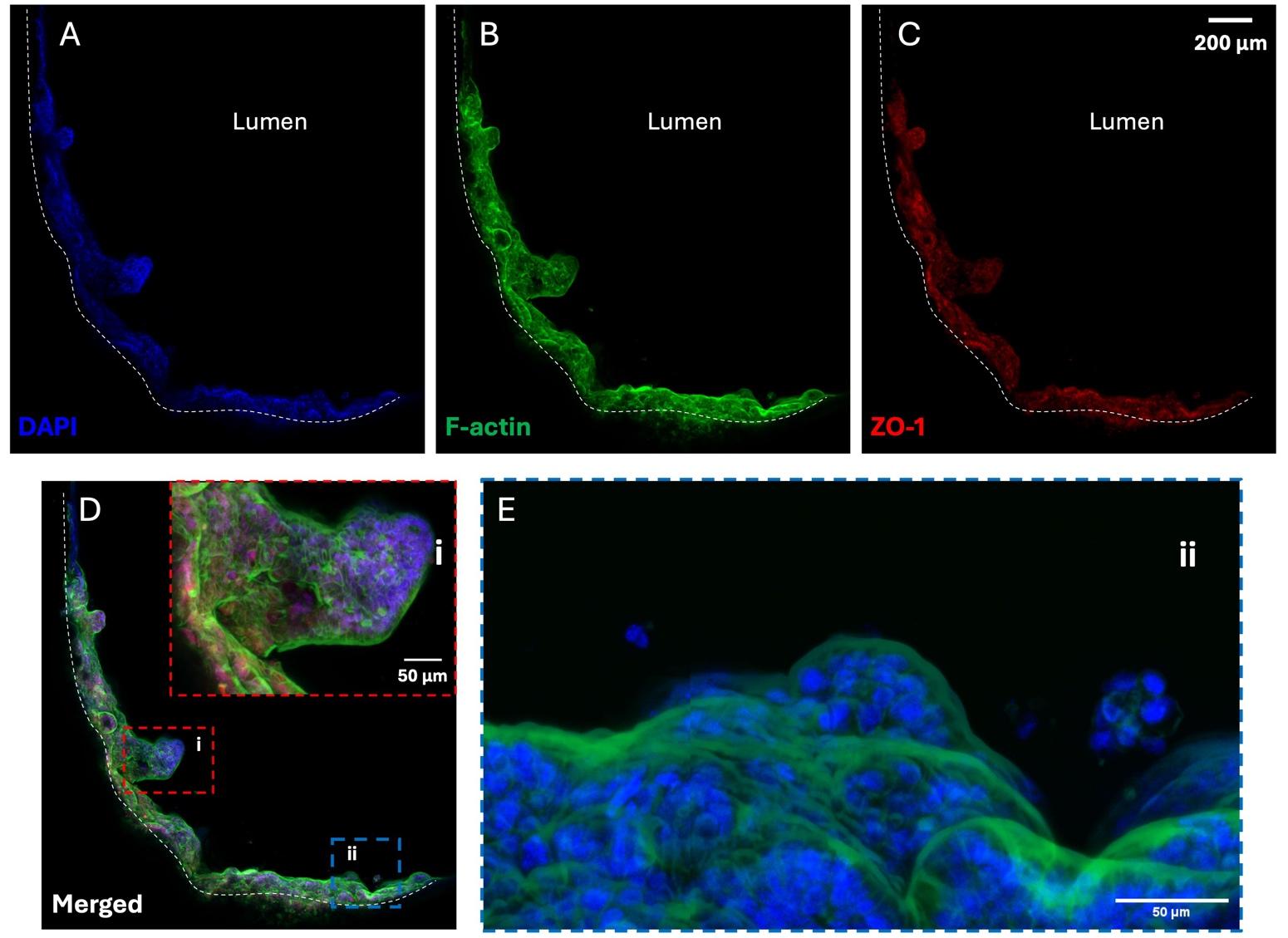


Fig. S8. Immunofluorescence characterization of intestinal epithelial differentiation in a 3D scaffold. A-C) Fluorescent staining of a cross-section of the scaffold shows the expression of ZO-1 (red), F-actin (green), and cell nuclei (blue), highlighting the establishment of a polarized epithelial layer. Scale bar: 200 μm. D) Merged image of the fluorescent markers within the scaffold, the quantitative assessment of epithelium height across the 3D model, has been measured with respect to the dashed white line presented. E) The inset provides a magnified view of the differentiated epithelial cells, illustrating the formation of villi and crypts. The images confirm the presence of tight junctions and the epithelial layer's compactness. Scale bar: 50 μm.

# Supporting Information Note 9 | Electrode Fabrication and biocompatibility assessment

Traditional methods to assess the functionality of the transepithelial barrier involve measuring the electrical resistance of cell monolayers cultivated in Transwell plates. However, scaling this measurement to a 3D bioprinted colon model presents a challenge due to the sample's size and fragility. To enable a proper comparison between traditional 2D and 2.5D tissue models, we designed and fabricated a scaled-up TEER (transepithelial electrical resistance) device. The electrode fabrication involved using a commercial gold-coated film cut into rectangles with a plotter cutter, based on the required dimensions. These rectangles were then rolled into cylinders of specific diameters. To secure the cylindrical shape and prevent contact with the copper layer, PDMS was applied as a coating agent (Fig. S9A). Two electrodes with different surface areas (1.25 cm² and 3.8 cm²) were used: external and internal electrodes in direct contact with the 3D bioprinted colon sample (Fig. S9B). The biocompatibility of these electrodes was assessed prior to their use with the colon samples. 3T3 fibroblasts were embedded in the bioink, the gold electrodes were formed, and the bioink was crosslinked to suspend the cells within the matrix. The cultures of cells in direct contact with the electrodes were evaluated for their effects on cell survival through metabolic activity measurements on days 1, 7, and 14. Results were normalized relative to day 1. Throughout the experiment, metabolic activity increased on days 7 and 14, indicating cell proliferation within the hydrogels and confirming the biocompatibility of the designed electrodes.


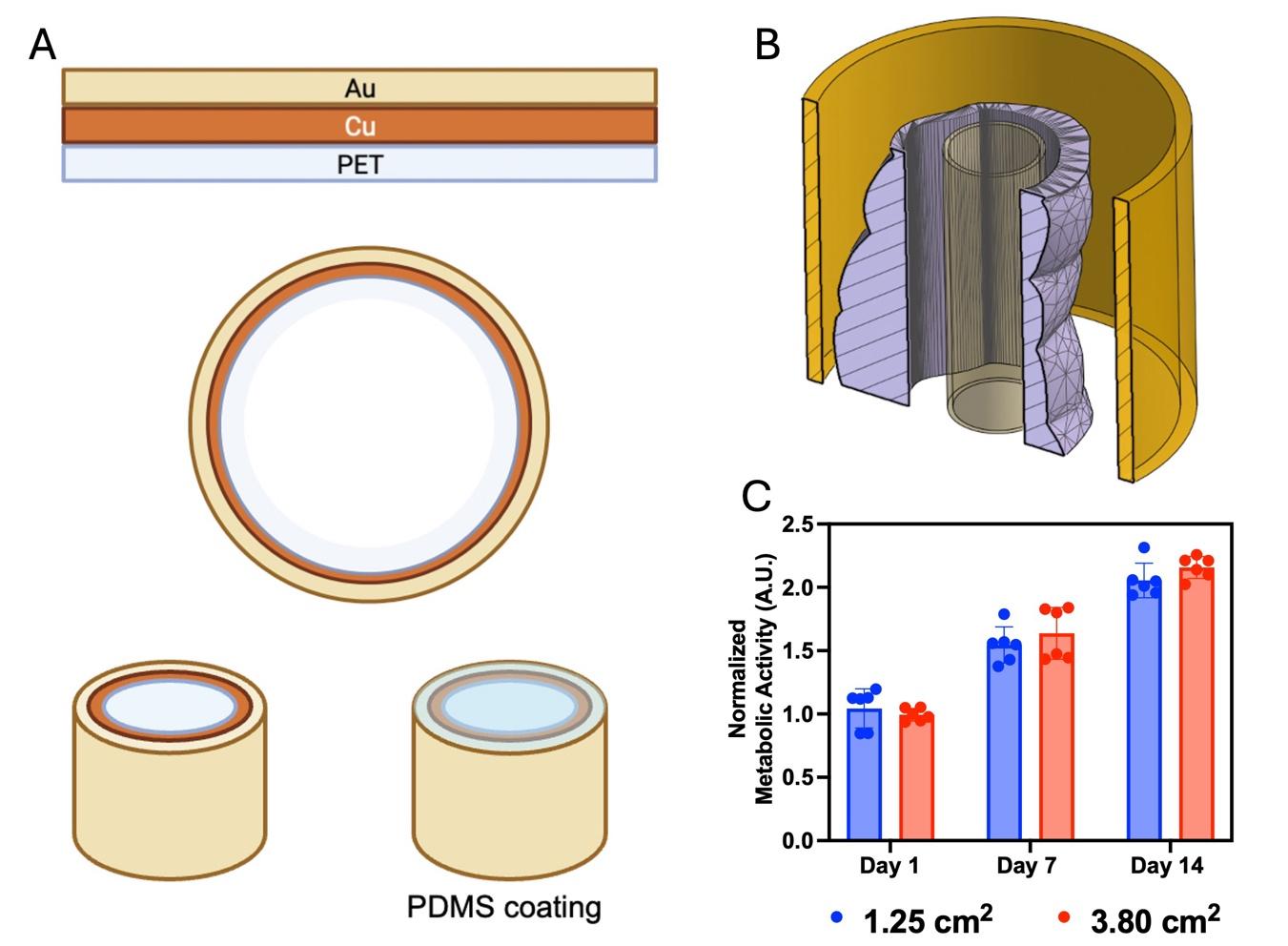


Fig. S9 Design and Fabrication of Gold Electrodes to Measure TEER in the 3D Bioprinted Colon.
A) Layer schematic of the gold film used to fabricate the electrodes. The commercial gold film consists of a PET substrate coated with 3 nm of copper, followed by a gold layer. The film was cut using a cutting plotter and rolled as a cylinder. To maintain structural integrity and prevent copper exposure to the culture medium, the lateral surfaces of the electrodes were coated with PDMS. B) Transversal representation of the gold electrode placement for TEER measurement in the 3D bioprinted colon, with the smaller electrode positioned at the center and the larger electrode surrounding it. C) Normalized metabolic activity of 3T3 fibroblast cells embedded in the bioink and cultured on gold electrodes of two different sizes (3.80 cm² and 1.25 cm²) over 14 days. Data are presented as mean ± standard deviation, obtained from six independent biological replicates, to evaluate the biocompatibility of the electrodes at days 1, 7, and 14.

# Supporting Information Note 10 | TEER chamber design and fabrication

To perform an accurate TEER measurement in the 3D bioprinted colon model and ensure uniform spacing between electrodes, a specialized chamber was designed to accommodate both the reference electrode (RE) and the working electrode (WE). Utilizing Solidworks (Solidworks Corp., USA), the chamber was conceptualized as a cylindrical structure with an inner diameter (DI) of 6 mm and a height of 10 mm. The upper section of the chamber serves as a lid and incorporates a central aperture designed to secure the second electrode, which has a DI of 4 mm. The design features a key-and-lock mechanism between the lid and the chamber body, ensuring stable positions for the electrodes throughout the measurement process. Details of the chamber's design, including specific dimensions, are provided in the supplementary materials (Figure S11). The fabrication process of the chamber involved 3D printing using a LumenX digital light processing printer (Cellink, USA) with plastic resin. The design was prepared for printing by slicing it into layers with a height of 100 µm. After printing, both the chamber and lid underwent thorough cleaning to remove any residual resin, followed by ultraviolet curing to ensure complete solidification.


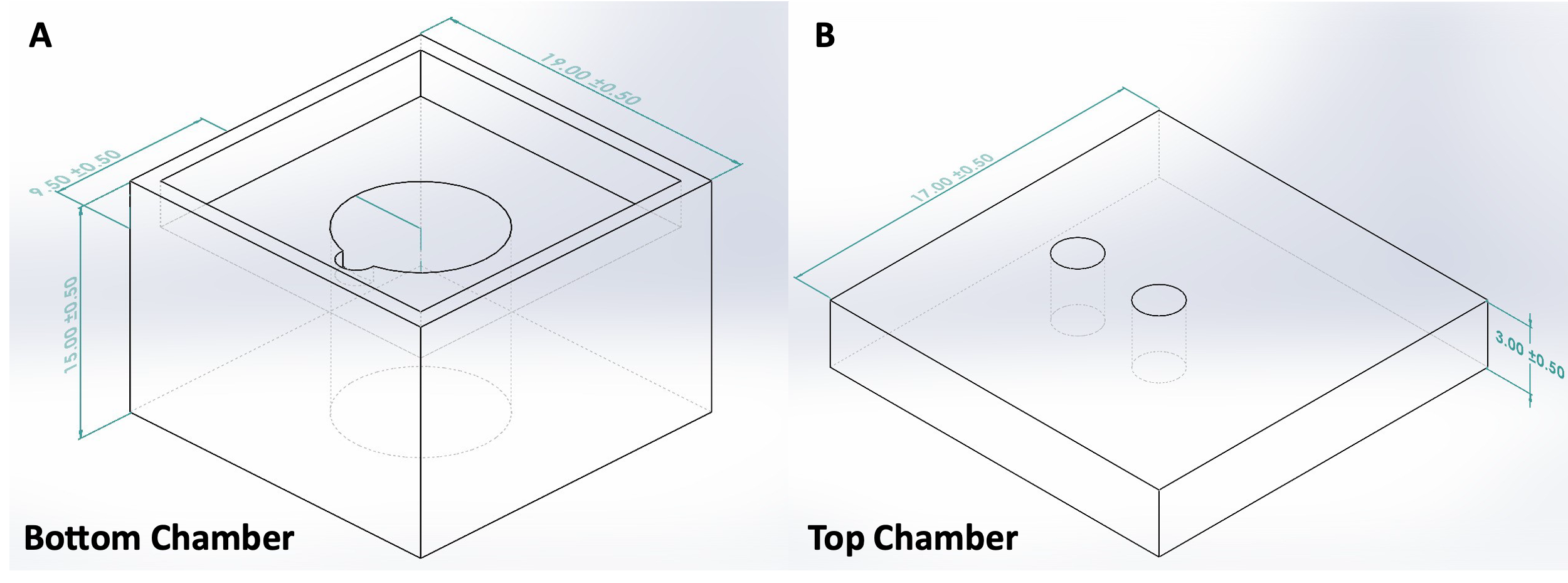


Fig. S10 Design of Electrode Chamber, bottom and top for TEER measurement in 3D colon constructs, assembled as key and lock configuration. (A) Bottom chamber design, (B) Top lid design.

# Supporting Information Note 11 | Simulation of Several Key Colonic Functions: Barrier Integrity, Water Absorption, and Nutrient Absorption

To complement our experimental evaluations and directly compare our 3D-printed colon model to native human tissue, we performed in silico simulations of three essential colonic functions: barrier integrity, water absorption, and nutrient absorption^2–4^. These functions were chosen because they are fundamental to overall colonic physiology. We have experimentally measured parameters (such as transepithelial electrical resistance (TEER), epithelial thickness, and crypt-like morphology) that support their accurate modeling^4,5^.

First, we generated a three-dimensional surface mesh of our 3D-printed construct’s epithelium by segmenting high-resolution immunostained confocal stacks and reconstructing crypt-like geometries. Autodesk Maya (Autodesk, USA) was used to import these image sets, clean up noise and artifacts, and produce a detailed surface mesh reflecting the epithelial topography and crypt-like domains. We then scaled the mesh to match human colonic dimensions while retaining the printed construct’s experimentally determined thickness (~20 µm for the epithelial layer) and diameter (~5 mm)^6^. Our printed construct is roughly 1/150 the length of an adult colon, whereas its diameter is scaled to ~1/10 of a typical human colon.

In this process, we kept the diameter and crypt dimensions proportionally consistent with the smaller form factor to maintain realistic surface topography. Rather than uniformly stretching or shrinking each crypt, we performed a length-based scaling of the luminal axis while preserving crypt curvature and approximate depth ratios. This approach ensured that crypt openings did not become unrealistically large or small relative to the rest of the construct^7^.

To run our finite element simulations, we used COMSOL Multiphysics (COMSOL Inc., USA) and generated a triangular surface mesh with approximately 100,000 elements capturing both the luminal surface and crypt invaginations. We did not add or remove elements during the scaling; instead, the same 3D mesh was mathematically scaled along the longitudinal axis. We integrated multiple software packages to ensure each function could be accurately represented: COMSOL Multiphysics was used to solve the convection–diffusion equations and partial differential equation (PDE) models related to flow, solute transport, and active absorption. Autodesk Maya assisted in the preparation and mapping of three-dimensional crypt topographies. MATLAB (MathWorks, USA) aided in pre-processing parameters, analyzing simulation results, and calculating error metrics (normalized root-mean-square error (NRMSE) and mean absolute percentage error (MAPE)) to compare simulated outcomes against human reference values. Throughout, we used a combination of direct experimental data and established gastrointestinal physiology references to parameterize the simulations. Each of the three functions (barrier integrity, water absorption, and nutrient absorption) was modeled with its own set of governing PDEs and boundary conditions, sharing a common geometric scaffold, flow fields, and measured TEER data. This means that while each function can be examined separately, they all rely on consistent parameters such as crypt geometry, epithelial thickness, and paracellular permeability.

**Barrier Integrity Simulation**

Barrier integrity is essential in the colon, determining paracellular permeability and protecting underlying tissues from pathogens. Published ex vivo and in vivo measurements of human colonic TEER indicate a wide range, reflecting differences in tissue handling, segment selection, and measurement protocol^2,8–11^. These studies also underscore how robust barrier function is critical for immune homeostasis and overall colonic health, as it prevents infiltration of luminal antigens, regulates fluid transport, and ensures proper nutrient absorption. Our laboratory measurements showed that TEER approached ~70 Ω·cm² in our construct, aligning with human colonic data. We used these TEER values directly in COMSOL to define the paracellular conductivity boundary condition for a simplified convection–diffusion–reaction PDE. The 3D surface mesh from confocal images was refined at crypt invaginations to capture localized solute gradients. A low-velocity laminar flow was imposed in the lumen, scaled to the reduced length of the printed colon. The model’s main goal was to compute how a small solute traverses the epithelial layer under measured TEER constraints and to quantify the flux relative to native colon permeability. Validation involved normalizing the flux by the measured crypt-resolved surface area, then comparing those fluxes to known colonic permeability data from the literatures. We found that the simulated construct matched ~99% of typical human colon values once the boundary conditions were tuned to reflect the experimentally obtained TEER and paracellular resistance.

**Water Absorption Simulation**

Water absorption is central to colonic function, as the colon reclaims fluid via osmotic and transporter-driven fluxes. We used COMSOL’s coupled convection–diffusion PDE, incorporating an osmotic gradient and paracellular resistance determined by TEER. Literature on human colonic fluid reabsorption shows a broad range (1–2 liters per day on average), with localized rates depending on factors like luminal ionic content and motility^3,12–14^.We applied a moderate flux target in our simulation to represent a typical adult colon segment, aligning with our scaled-down geometry and the measured TEER constraints. The same geometry was retained, but lumen volumes and flow rates were set to typical colonic fluid ranges (scaled to our 1:150 ratio). The paracellular permeability was fixed at the same conductivity derived from TEER measurements to maintain consistency. Because direct fluid uptake was not measured in real time, we compared the model’s net predicted flux (in µL/cm² per hour) to established data and meta-analyses for adult colon absorption^3,12–14^. The outcome matched ~80% of native capacity. This value was consistent with the TEER-based constraints, the partial presence of aquaporins and transporters in our epithelium, and our simplified luminal fluid properties.

**Nutrient Absorption Simulation**

Although we did not measure specific nutrient flux experimentally, nutrient absorption modeling provides insight into how crypt topography and paracellular resistance influence uptake. We again used COMSOL with a convection–diffusion–reaction module. Transcellular transporter-mediated uptake was modeled using Michaelis–Menten kinetics, with Km and Vmax values taken from colon epithelial literature ^13,15–18^. Observed human colonic nutrient uptake rates can fluctuate based on diet, luminal pH, and regional transporter expression. We used an intermediate uptake scenario (roughly matching midrange carbohydrate and amino acid absorption reported ex vivo), consistent with our measured crypt geometry and barrier function. We maintained the same crypt-resolved geometry and set local flow velocities from our barrier integrity simulations. The simulation aimed to estimate the steady-state nutrient absorption rate normalized by surface area. We then compared the predicted uptake to ex vivo human colon data on carbohydrate or amino-acid absorption. The final result converged at ~75% efficiency compared to published rates. We interpret this as a reasonable compromise given the reduced construct scale, partially optimized nutrient transporters, and a less complex luminal microenvironment than in vivo.

Our simulations showed that each of the three targeted functions was reproduced at about 75% to 99% of native human colon levels (Fig. S10). All three were modeled in separate PDE frameworks but relied on the same geometric mesh and boundary conditions, ensuring internal consistency. These outcomes are grounded in experimentally derived parameters. In all simulations, the main geometry files (3D meshes from confocal images), boundary condition spreadsheets, PDE model definitions, and source-code scripts for COMSOL and Autodesk Maya are available upon request. By focusing on three major functions supported by robust experimental data, our simulation framework demonstrates that the 3D-printed colon construct recapitulates several critical aspects of native colonic physiology and thus offers a validated platform for further disease modeling and drug-response studies.

# Supporting Information Note 12-13 | Cancer spheroid fabrication and characterization

The formation of HCT116 carcinoma spheroids was conducted using the hanging drop method (Fig. S11). A known concentration of cells dispersed in the medium was placed in a 20 µL droplet on the lid of a petri dish. The lid was then inverted to create hanging drops, and 2 mL of PBS was added to the petri dish to prevent media evaporation. The petri dish was incubated for 7 days, resulting in well-formed spheroids. We performed an exploratory study to identify which cell concentration (ranging from 10 × 10⁵ to 50 × 10⁵ cells) would result in consolidated spheroids with diameters of approximately 500 µm after 7 days of culture (Fig. 11B). On Day 1, initial cell aggregation was observed across all four concentrations. However, by Days 3 and 7, spheroids formed with cell concentrations of 40 × 10⁵ and 50 × 10⁵ exhibited a more pronounced and uniform round shape. Spheroids grew in size and became denser from Day 1 to Day 7, with higher cell concentrations producing more cohesive and spherical structures.


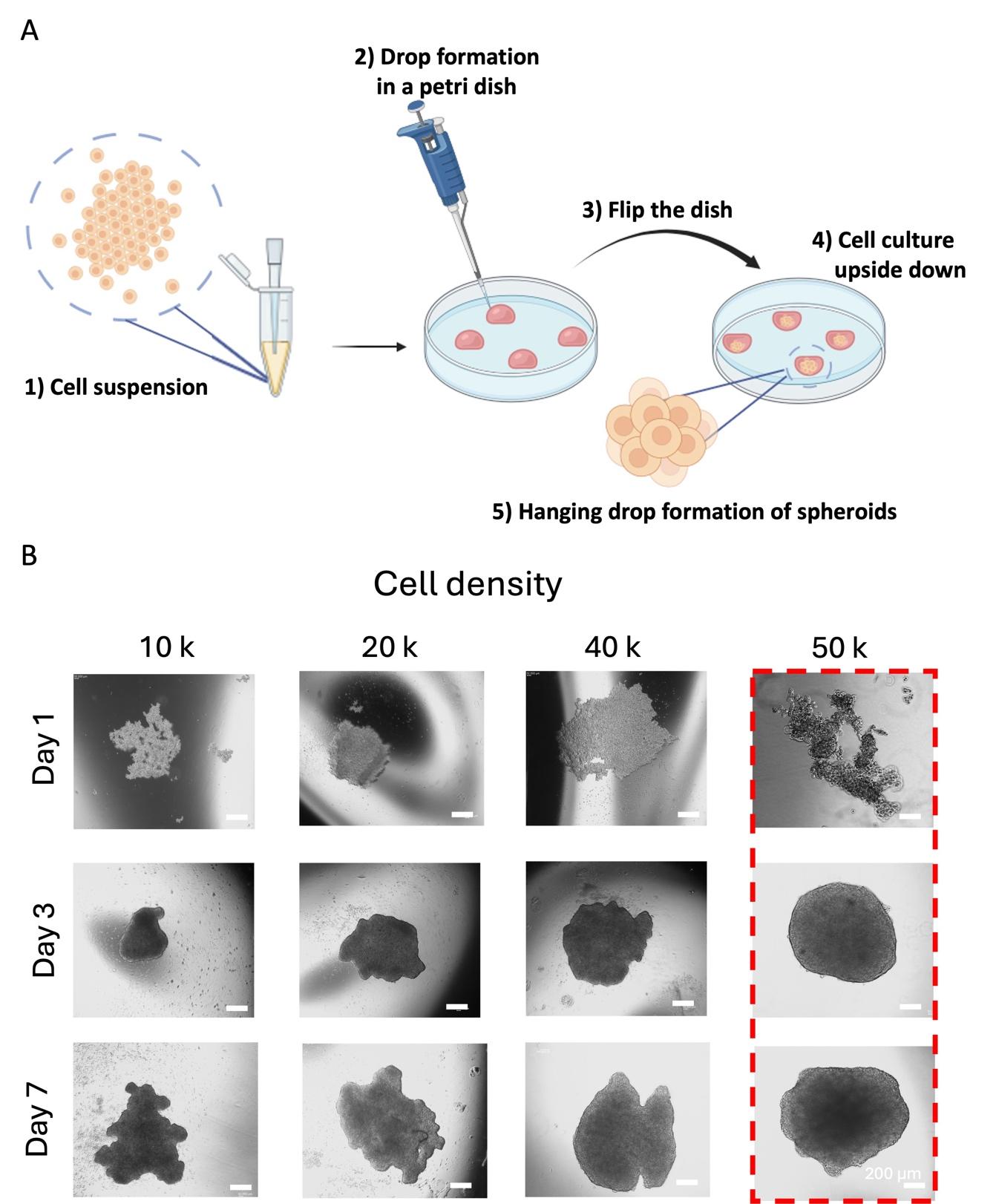


Fig. S11 Formation and growth progression of HCT116 spheroids via the hanging-drop method. A) Schematic representation of the hanging drop technique for spheroid formation. 1) A cell suspension is prepared, 2) small droplets of the suspension are pipetted onto the lid of a petri dish, 3) the lid is inverted, and 4) the cells aggregate under gravity to form spheroids within the hanging drops. B) Microscopy images showing the effect of initial cell density (10k, 20k, 40k, and 50k cells per droplet) on spheroid formation over time (Day 1, Day 3, and Day 7). Scale bar: 200 μm

After 7 days of maturation, the spheroids were carefully transferred into the lumen of the 3D bioprinted colon structures and positioned in direct contact with the epithelial layer to mimic the presence of cancer tumors in the colon. Two spheroids were seeded per colon, and brightfield microscopy was used to monitor their position and integration into the colon wall over three days in preparation for drug testing assays as shown in Fig S12.


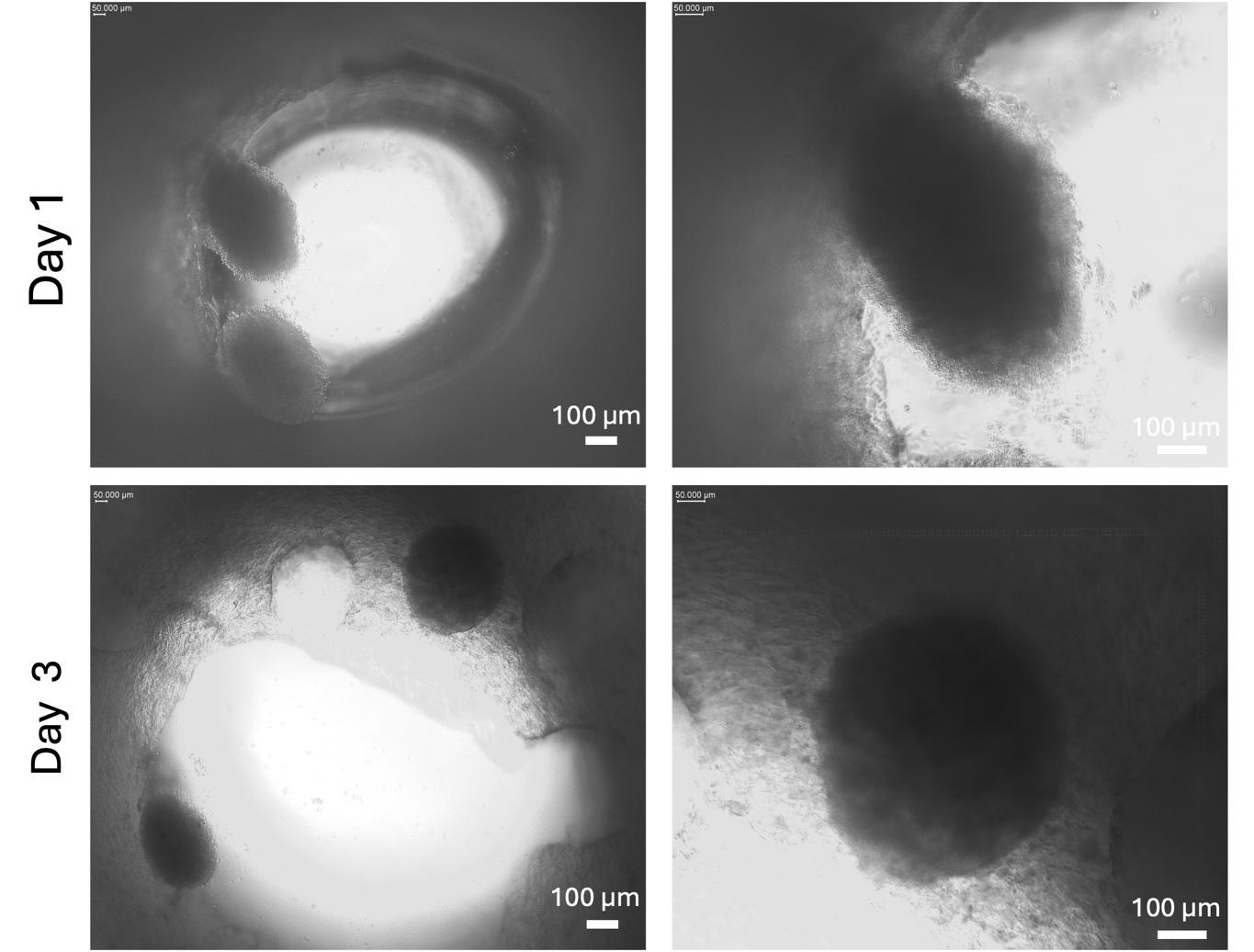


Fig. S12 Brightfield micrographs of HCT116 adenocarcinoma spheroids seeded onto the epithelial layer of the 3D bioprinted colon, observed on Days 1 and 3. Scale bar: 200 μm.

# Supporting Information Note 14 | Drug efficacy studies


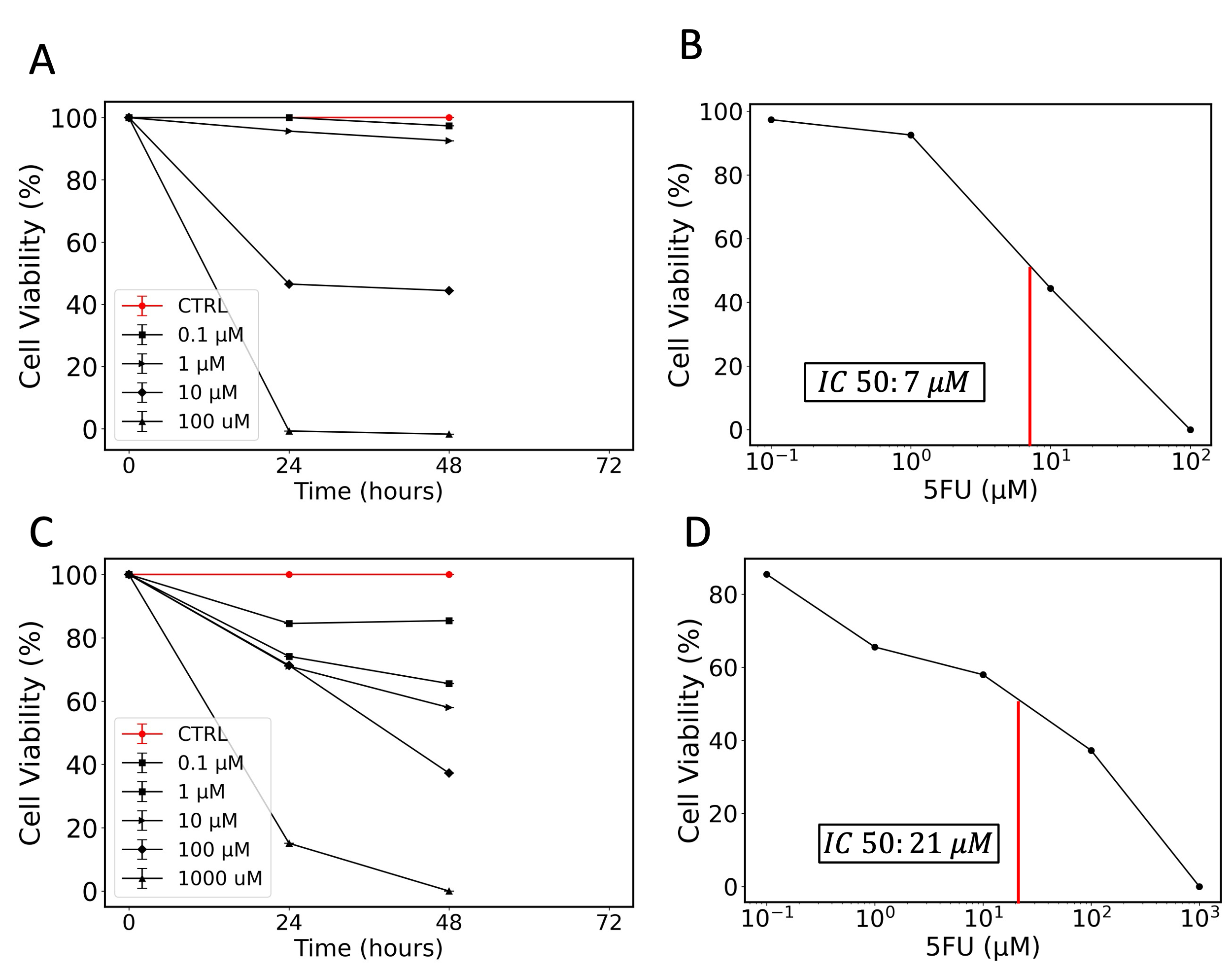


Fig. S 13 Drug efficacy study for HCT116 cell monoculture and 3D spheroid in well plate format. (E) Dose-dependent cell viability of the HCT116 monoculture over time upon 5FU treatment at 0.1-1-10-100 µM. (F) Determination of IC50 value for the HCT116 monoculture by non-linear regression. (G) Cell viability of the 3D spheroid model over time with varying concentrations of 5FU, at 0.1-1-10-100-1000 µM. (H) IC50 value estimation for the 3D spheroid model, indicating a significant increase compared to the monoculture model.

**References**

1. Tavares-Negrete, J. A. *et al.* Three-Dimensional Printing Using a Maize Protein: Zein-Based Inks in Biomedical Applications. *ACS Biomater. Sci. Eng.* **7**, 3964–3979 (2021).

2. Groschwitz, K. R. & Hogan, S. P. Intestinal barrier function: Molecular regulation and disease pathogenesis. *Journal of Allergy and Clinical Immunology* **124**, 3–20 (2009).

3. Rao, M. C., Sarathy, J. & Ao, M. Intestinal Water and Electrolyte Transport in Health and Disease. *Colloquium Series on Integrated Systems Physiology: From Molecule to Function* **4**, 1–105 (2012).

4. González-Quilen, C. *et al.* Protective properties of grape-seed proanthocyanidins in human ex vivo acute colonic dysfunction induced by dextran sodium sulfate. *Eur J Nutr* **60**, 79–88 (2021).

5. Srinivasan, B. *et al.* TEER Measurement Techniques for In Vitro Barrier Model Systems. *SLAS Technology* **20**, 107–126 (2015).

6. Montes-Olivas, S. *et al.* In-silico and in-vitro morphometric analysis of intestinal organoids. *PLoS Comput Biol* **19**, e1011386 (2023).

7. Mehl, L. E. A mathematical computer simulation model for the development of colonic polyps and colon cancer. *Journal of Surgical Oncology* **47**, 243–252 (1991).

8. Vancamelbeke, M. & Vermeire, S. The intestinal barrier: a fundamental role in health and disease. *Expert Review of Gastroenterology & Hepatology* **11**, 821–834 (2017).

9. Adhikari, J. *et al.* Effects of Processing Parameters of 3D Bioprinting on the Cellular Activity of Bioinks. *Macromolecular Bioscience* **21**, 2000179 (2021).

10. Watson, C. J., Hoare, C. J., Garrod, D. R., Carlson, G. L. & Warhurst, G. Interferon-γ selectively increases epithelial permeability to large molecules by activating different populations of paracellular pores. *Journal of Cell Science* **118**, 5221–5230 (2005).

11. Urciuolo, F., Imparato, G. & Netti, P. A. Engineering Cell Instructive Microenvironments for In Vitro Replication of Functional Barrier Organs. *Adv Healthcare Materials* **13**, 2400357 (2024).

12. Binder, Henry & Sandle, Geoffrey. Electrolyte Absorption and Secretion in the Mammalian Colon. in *Physiology of the Gastrointestinal Tract* vol. 2 (Elsevier Academic Press, 1987).

13. Ferraris, R. P. & Diamond, J. Regulation of intestinal sugar transport. *Physiological Reviews* **77**, 257–302 (1997).

14. Walton, K. D., Freddo, A. M., Wang, S. & Gumucio, D. L. Generation of intestinal surface: an absorbing tale. *Development* **143**, 2261–2272 (2016).

15. Artursson, P., Palm, K. & Luthman, K. Caco-2 monolayers in experimental and theoretical predictions of drug transport. *Advanced Drug Delivery Reviews* **64**, 280–289 (2012).

16. Pappenheimer, J. R. Physiological regulation of transepithelial impedance in the intestinal mucosa of rats and hamsters. *J. Membrain Biol.* **100**, 137–148 (1987).

17. Drozdowski, L. Intestinal sugar transport. *WJG* **12**, 1657 (2006).

18. Johnson LR. *Gastrointestinal Physiology*. (Elsevier Health Sciences, 2013).
